# Reprogramming human gallbladder cells into insulin-producing β-like cells

**DOI:** 10.1371/journal.pone.0181812

**Published:** 2017-08-16

**Authors:** Feorillo Galivo, Eric Benedetti, Yuhan Wang, Carl Pelz, Jonathan Schug, Klaus H. Kaestner, Markus Grompe

**Affiliations:** 1 Oregon Stem Cell Center, Papé Family Pediatric Research Institute, Oregon Health & Science University, Portland, Oregon, United States of America; 2 Department of Genetics, School of Medicine and Institute of Diabetes, Obesity, and Metabolism, University of Pennsylvania, Philadelphia, Pennsylvania, United States of America; International University of Health and Welfare School of Medicine, JAPAN

## Abstract

The gallbladder and cystic duct (GBCs) are parts of the extrahepatic biliary tree and share a common developmental origin with the ventral pancreas. Here, we report on the very first genetic reprogramming of patient-derived human GBCs to β-like cells for potential autologous cell replacement therapy for type 1 diabetes. We developed a robust method for large-scale expansion of human GBCs *ex vivo*. GBCs were reprogrammed into insulin-producing pancreatic β-like cells by a combined adenoviral-mediated expression of hallmark pancreatic endocrine transcription factors *PDX1*, *MAFA*, *NEUROG3*, and *PAX6* and differentiation culture *in vitro*. The reprogrammed GBCs (rGBCs) strongly induced the production of insulin and pancreatic endocrine genes and these responded to glucose stimulation *in vitro*. rGBCs also expressed an islet-specific surface marker, which was used to enrich for the most highly reprogrammed cells. More importantly, global mRNA and microRNA expression profiles and protein immunostaining indicated that rGBCs adopted an overall β-like state and these rGBCs engrafted in immunodeficient mice. Furthermore, comparative global expression analyses identified putative regulators of human biliary to β cell fate conversion. In summary, we have developed, for the first time, a reliable and robust genetic reprogramming and culture expansion of primary human GBCs—derived from multiple unrelated donors—into pancreatic β-like cells *ex vivo*, thus showing that human gallbladder is a potentially rich source of reprogrammable cells for autologous cell therapy in diabetes.

## Introduction

Type 1 Diabetes Mellitus (T1DM) is a metabolic disorder characterized by hyperglycemia caused by autoimmune-mediated destruction of pancreatic β cells leading to a complete or near total loss of the hormone insulin [1, 2]. Daily administration of exogenous insulin is still the mainstay of treatment, but this is not always sufficient to prevent chronic complications of the retina, kidneys, peripheral nerves, and blood vessels. Replacement of β cells (either by whole pancreas or islet cell transplantation) is the best available method for restoring a physiologic glycemic control and improvement of diabetes complications [3, 4]. As with other types of organ transplantation, shortcomings related to islet or whole pancreas transplantation include the high medical and surgical costs, insufficient supply of available donor pancreata, and lifetime requirement for immunosuppression therapy—that is variably associated with adverse side-effects [3, 5–7]. Therefore, alternative sources of abundant transplantable β cells are needed to fill the supply gap [8–15].

Potentially, there are two general approaches to generate β cell replacements: (a) stage-wise *in vitro* differentiation of pluripotent stem cells (PSCs) using extrinsic protein factors and small molecules [11–16], and (b) reprogramming of adult cells from endoderm-derived tissues by ectopic expression of pancreatic endocrine transcription factors [10, 17–23]. Recently, several published reports [11, 14, 15] have shown significant advancements in the *in vitro* differentiation of human PSCs into a mature β cell phenotype by efficiently recapitulating pancreatic endocrine development better than previous studies [8, 12, 16, 24]. In spite of achieving abundant functional β-like cells, the clinical usefulness of PSC-derived β cells may still be hampered by risk of tumor formation, immunogenicity and epigenetic abnormalities [25, 26]. On the other hand, multiple adult cell types had been directly reprogrammed towards the β cell fate including hepatocytes [18, 21, 23, 27, 28], pancreatic exocrine cells [20, 22, 29], intrahepatic biliary cells [19, 30], amniotic fluid cells [9], adipocytes [31, 32], antral gastric cells [33], and fibroblasts [18]. The transdifferentiation potential of these cell types could be influenced by epigenetic memory of their respective tissue of origin [26] which may predispose a higher degree of β cell reprogramming for endodermal derivatives than cells from other germ layers [18].

Based on the common developmental origin of the ventral pancreas, the liver and its associated biliary tree from the posterior ventral foregut [34] and from reports of ectopic pancreatic tissues found in extrahepatic biliary tree [35–37], our group previously showed that murine gallbladder can be dependably reprogrammed into insulin-producing islet-like cells after forced expression of *Pdx1*, *MafA*, and *Neurog3 ex vivo* [10, 38]. Here, we embarked on the very first reprogramming, from multiple donors, of human primary gallbladder and cystic duct cells. The overarching aim of this study was to genetically reprogram highly expandable patient-derived human gallbladder cells (GBCs) to produce insulin for potential autologous transplant in T1DM patients in the future. The gallbladder and cystic duct are attractive sources of potentially renewable β-like cells not only for their close developmental origin to the pancreas, but also for their amenability for surgical extirpation without serious adverse health effects [10]. In this study, we developed a reliable method for the large-scale expansion of patient-derived human GBCs *ex vivo* for the purpose of genetic reprogramming to β cell fate. We reprogrammed GBCs into insulin-producing pancreatic β-like cells by combining adenoviral mediated transduction of pancreatic endocrine transcription factors *PDX1*, *MAFA*, *NEUROG3*, and *PAX6* and small molecule-dependent transdifferentiation. Global mRNA and microRNA expression profiles and protein immunostaining experiments indicate that reprogrammed GBCs (rGBCs) adopted an overall β-like state and could transiently engraft in immunodeficient recipients.

## Materials and methods

### Extrahepatic biliary tissue specimens

Normal human gallbladder and cystic duct specimens (collectively referred to as extrahepatic biliary tissues or EHBT) were obtained from the Department of Surgical Pathology at the Oregon Health & Science University (OHSU). The pathologist/research pathology technician-on-duty selected and excised grossly normal EHBT fragments (1 cm^3^ to 3 cm^3^) and kept them on ice in DMEM/F12 supplemented with 100 units/mL Penicillin and 100 μg/mL Streptomycin (Mediatech, Manassas, VA, USA). Tissue samples were anonymized as outlined in the IRB study exemption approved by the OHSU Institutional Review Board. Single cell suspensions of gallbladder and cystic duct (collectively referred to as gallbladder and cystic duct cells or GBCs) were prepared by protease digestion. First the tissue (EHBT) was incubated in 1X EBSS (Gibco, Grand Island, NY, USA) supplemented with 10 mM EGTA (Sigma, St. Louis, MO, USA) and 1% v/v HEPES buffer (Mediatech, Manassas, VA, USA) for 5 minutes at room temperature. The EHBT fragments were then digested in 5 mg/mL collagenase II (Gibco, Grand Island, NY, USA) in 1X HBSS (HyClone Laboratories, South Logan, UT, USA), 20 μg/mL DNaseI (Roche, Laval, QC, Canada) and 1% v/v HEPES (Mediatech, Manassas, VA, USA) for a total of 2-hours with intermittent agitation in a 37^°^C water bath. EHBT tissue chunks were subsequently strained through a 100 μm nylon mesh (BD Falcon, Bedford, MA, USA) and the resulting suspension of GBCs was washed and treated with ACK lysis solution (0.15M NH _4_Cl, 10 mM KHCO_3_, 0.1 mM EDTA, pH 7.4, 0.22 μm filter-sterilized) for 2 minutes at room temperature to remove red blood cells. Finally, GBCs were washed and strained using a 40 μm nylon mesh (BD Falcon, Bedford, MA, USA) and suspended in DMEM/F12 supplemented with 0.5% fetal bovine serum (Sigma-Aldrich, St. Louis, MO, USA) and kept on ice. To archive tissues, EHBT samples were cut into less than 1 cm^3^ fragments and embedded in Tissue-tek cryomatrix (Sakura, Tokyo, Japan) and stored at -81^°^C.

### Culture and expansion of primary human extrahepatic biliary cells

Primary cultures of GBCs were initiated as a modification of previously reported protocol [10, 39]. LA7-RSPO1 rat mammary tumor cells (stably expressing human R-Spondin-1 via lentiviral transduction and puromycin selection) were used as feeder cells after irradiation (6 Gy), plated in plastic wells (1,200 cells/mm ^2^), and grown for at least 4 hours in 37^°^C and humidified 5% CO_2_ incubator. Dispersed GBCs were obtained by collagenase digestion as outlined above and resuspended in GBC growth medium: 1X DMEM/F12 (Gibco, Grand Island, NY, USA), 0.5% fetal bovine serum v/v, 100 Units/mL Penicillin and 100 μg/mL Streptomycin, 1X GlutaMAX ^TM^ (Gibco, Grand Island, NY, USA), 1X MEM Non-essential amino acids (Mediatech, Manassas, VA, USA), 15 mM HEPES buffer, 1X Insulin-Transferrin-Sodium Selenite (Roche Diagnostics, Indianapolis, IN, USA), 200 ng/mL cholera toxin (Sigma, St. Louis, MO, USA), 1.25 mM N-acetyl-L-cysteine (Sigma, St. Louis, MO, USA), and 5 mM nicotinamide (Sigma, St. Louis, MO). GBCs were plated on top of irradiated LA7-RSPO1 and allowed to attach for 48 hours prior to media change. GBCs were fed every 2–3 days and repassaged onto a fresh feeder layer upon reaching 90% cell confluence.

### Viral transduction and reprogramming

CMV promoter-driven replication-defective adenoviruses serotype 5 (Ad5) with deletion of both E1 and E3 genes were used. Monocistronic Ad5 individually encoded PAX6, NEUROG3, PDX1, MAFA, or GFP and multicistronic Ad5 vectors included NPM (NEUROG3, PDX1, MAFA) and M6P-GFP (MAFA, PAX6, PDX1, GFP) [17]. We harvested confluent cultures of GBCs by washing with 1X HBSS followed by incubation with 1X EBSS with 10 mM EGTA and 1% HEPES buffer for 5 minutes at 37^°^C. GBCs were detached using Accutase (Innovative Cell Technologies, Inc, San Diego, CA, USA) for 15–20 minutes and diluted in 0.5% fetal bovine serum in 1X DMEM/F12 and transferred into a conical tube. GBCs were pelleted by centrifugation at 700 rpm for 7 minutes at 4^°^C. For adenoviral transduction, CsCl-purified adenoviruses (MOI = 250–500 vg/cell ~ 1–2 pfu/cell per virus type) were added directly into the cell pellets and finally resuspended in 2% FBS in 1X DMEM/F12 (~250 μL/5x10^6^ cells). The cell-virus suspensions were incubated on ice for 2 hours with intermittent agitation every 15–20 minutes. Transduced cells were washed once with 1X DMEM/F12, resuspended in GBC growth medium and dispensed drop-wise (5,000 cells/mm^2^) into culture vessels pre-incubated with ~500–700 μL of Advanced DMEM/F12 medium with 10% Geltrex^TM^ LDEV-free, hESC-qualified, reduced growth factor (Gibco, Grand Island, NY, USA). Plated cells were incubated overnight in a 37^°^C, humidified 5% CO_2_ incubator. The next day, culture medium was replaced with reprogramming medium 1 (RM1): 1x Advanced-DMEM/F12 (Gibco, Grand Island, NY, USA), 0.5% fetal bovine serum v/v, 100 Units/mL Penicillin and 100 μg/mL Streptomycin, 1X GlutaMAX ^TM^, 1X MEM Non-essential amino acids, 15 mM HEPES buffer, 1X Insulin-Transferrin-Sodium Selenite, 1.25 mM N-Acetyl-L-cysteine, 5 mM nicotinamide, and 0.33 μM retinoic acid (Sigma, St. Louis, MO, USA). RM1 was applied for four days. Basal maturation medium (MM) was used starting on the 5th day until the last day of reprogramming on day 14 and had the following components:1x High-glucose DMEM, 0.5% fetal bovine serum v/v, 100 Units/mL Penicillin and 100 μg/mL Streptomycin, 1X GlutaMAX ^TM^, 1X MEM non-essential amino acids, 15 mM HEPES buffer, 1X insulin-transferrin-sodium selenite, 1.25 mM N-acetyl-L-cysteine, and 5 mM nicotinamide. MM1 was used on day 5 and 6 and consisted of MM supplemented with 0.33 μM retinoic acid, 1X B-27 supplement (Gibco, Grand Island, NY, USA), 55 nM human GLP-1 (Sigma, St. Louis, MO, USA), 25ng/mL rhKGF (BioLegend, San Diego, CA, USA), 50 ng/mL rhFGF-10 (Invitrogen, Carlsbad, CA, USA), 1% Geltrex (GIBCO, Grand Island, NY, USA), 0.1 mM LDN193189 (Sigma, St. Louis, MO, USA), 0.25 mM SANT-1 (Cayman Chemical Company, Ann Arbor, MI, USA). On days 7–9, culture medium was changed to MM2 (MM1 + 25 ng/mL EGF (GIBCO, Grand Island, NY, USA) + 0.25 mM L-Ascorbic Acid (Sigma, St. Louis, MO, USA)). From days 10–14, reprogramming medium was shifted to MM3 (MM + 1X B-27 (GIBCO, Grand Island, NY, USA) + 55 nM GLP-1 + 10 μM DAPT (TOCRIS Bioscience, Avonmouth, Bristol, UK) + 40 μM ISX-9 (Cayman Chemical Company, Ann Arbor, MI, USA) + 50 μg/mL heparin (Sigma, St. Louis, MO, USA) + 0.1 mM LDN + 0.25 mM L-Ascorbic Acid + 1% Geltrex).

### RNA isolation, cDNA synthesis and quantitative PCR

Reprogrammed GBCs (rGBCs) were washed with 1X PBS once and Trizol reagent was directly added to the cells. RNA was isolated following the Trizol standard protocol. For cDNA synthesis, 1–5 μg of RNA was reverse transcribed using M-MLV reverse transcriptase (Invitrogen, Carlsbad, CA, USA) following the manufacturer’s recommended protocol in the presence of 25 μg/mL oligo(dT)18, 0.5 mM dNTP, 10 mM DTT, 40 units RNaseOUT recombinant ribonuclease inhibitor (Invitrogen, Carlsbad, CA, USA). SYBR-based quantitative PCR was performed by utilizing either the iQ^TM^ 5 or CFX96 Touch^TM^ Real-Time PCR Detection System (Bio-Rad, Hercules, CA, USA) to measure gene expression. The qPCR reactions were comprised of Platinum^®^ Taq polymerase, 2.5 mM MgCl_2_, 10 μM DNA primers, 10 mM dNTPs, and 0.5X SYBR green (Invitrogen, Carlsbad, CA, USA). Thermocycling reactions were run as follows: 45 cycles of 15 s at 95^°^C, 20 s at 68^°^C, and 25 s at 72^°^C. PCR primers are shown in S1 Table.

### Measurement of intracellular and secreted c-peptide

For measurement of intracellular of C-peptide content, we harvested two-week old rGBC by rinsing with prewarmed PBS 3 times and lysing in 1X cell lysis buffer (Cell Signaling Technology, Danvers, MA, USA) with a complete mini-protease inhibitor cocktail (Roche, Mannheim, Germany). The cells were scraped off the dish, sonicated, and centrifuged at 14,000g at 4^°^C, saving the supernatant for C-peptide ELISA (Mercodia, Uppsala, Sweden). The C-peptide secretion assay was done as described previously [17]. Specifically, 2-week old rGBC in 12-well plates were incubated in fresh prewarmed day 14 reprogramming medium (MM3) for 2 hours at 37^°^C, then followed by rinsing three times with plain Krebs-Ringer bicarbonate buffer (KRBB) 20 minutes each at 37^°^C. Subsequently, rGBC were incubated with 150 μL KRBB with or without 1mM or 2.8 mM glucose for 2 hours in a humidified 37^°^C incubator. Supernatants were collected, centrifuged to remove cell debris, and stored at -80^°^C. Into the same wells, we added 150 μL KRBB with 25 mM glucose and incubated for 2 additional hours and harvested the supernatants. Total protein contents were measured using a BCA protein assay kit (Pierce Biotechnology, Rockford, IL, USA). Glucose-stimulated C-peptide was measured using Ultrasensitive Human C-peptide ELISA kit (Mercodia, Uppsala, Sweden).

### Flow cytometry

Dissociated GBCs were incubated in Hpi1 or Hpi2 mAb (courtesy of Oregon Stem Cell Antibody Core) [40] for 30 min at 4^°^C and washed with cold DMEM prior to labeling with APC-conjugated anti-mouse IgG (Jackson ImmunoResearch, West Grove, PA, USA). Propidium iodide (10 μg/mL) (Molecular Probes, Eugene, OR, USA) was included to mark dead cells. Cells were analyzed with a FACSCalibur or sorted by Influx-GS (BD Biosciences, San Jose, CA, USA) at 15 psi using a 100 μm nozzle. FlowJo software (Treestar, Ashland, OR, USA) was used to analyze flow cytometric data.

### RNA sequencing

FACS-sorted rGBC were subdivided based on positive staining with the Hpi1 mAb [40] and collected directly into a 1.5 mL microcentrifuge tube with 750 μL Trizol-LS. RNA was isolated using a modified Trizol-LS protocol and subsequent column-based method using Qiagen RNeasy mini kit (Qiagen GmbH, Hilden, Germany). RNAseq libraries were constructed and indexed using TruSeq RNA library prep kit v2 (Illumina Inc., San Diego, CA, USA). Short read sequencing assays were performed by the OHSU Massively Parallel Sequencing Shared Resource using Illumina HiSeq2000. RNA sequencing data are available from ArrayExpress database (accession number E-MTAB-5556). RNA library sequencing reads were aligned and annotated according to UCSC RefSeq (hg19 assembly). We used START Shiny App (https://kcvi.shinyapps.io/START) [41] for pairwise comparisons and statistical calculations of p-values and adjusted p-values. Absent or lowly expressed genes in all samples (RPKM<1) were not included. Differentially expressed genes were picked based on log_2_ FC>1 and *p*<0.01. We further selected differentially expressed genes (log_2_ FC>5 and *p*<0.01) for gene set enrichment analyses (GSEA) [42]. Heat maps and hierarchical clustering (one-minus Pearson correlation or Euclidian distance) were generated using the GENE-E or Morpheus online tool (Broad Institute, MIT) and graphs were made using Prism 7 (GraphPad Software, Inc., La Jolla, CA, USA). Venn diagrams were generated using Pacific Northwest National Laboratory Venn Diagram Plotter.

### microRNA sequencing

Total RNA from FACS-sorted and unsorted rGBC and untransduced GBC were isolated using Qiagen microRNeasy minikit (Qiagen GmbH, Hilden, Germany) following the manufacturer’s protocol. microRNA libraries were generated and processed by the University of Pennsylvania’s Next Generation Sequencing Core using Illumina truSeq small RNA library preparation kit (Illumina, San Diego, CA, USA) configured for 50 cycle and sequenced on Illumina HiSeq 2500 (Rapid-V2). MicroRNA sequencing data are available from ArrayExpress database (accession number E-MTAB-5554). Adapter sequence was trimmed from the 3’-end of the sequences allowing up to 1 mismatch every 4 bases. Trimmed reads were aligned against premiRNAs from mirBase18 using Bowtie. Only reads with a single best alignment were considered. Trimmed reads aligned to premiRNA were checked for overlap with annotated or inferred mature forms (miRbase v18). We compared rGBC and GBC with publicly accessed datasets on pancreatic β cells [43] (cell.beta dataset GEO accession number GSM1262771). We calculated differential expression by using a simple fold change MvA comparison analysis (where M = log_2_ rGBC/β fold change and A = log_2_ rGBC average expression). We selected microRNAs with RPM>5 and FC>10 as differentially expressed. Heat maps, Venn diagrams, and graphs were created similar to RNA sequencing analyses.

### Transplantation of reprogrammed gallbladder cells

All animal experiments were done at OHSU and approved by the OHSU Institutional Animal Care and Use Committee. Transplantation experiments involved the use of 8–12 week old NSG (NOD.Cg-Prkdc^scid^Il2rg^tm1Wjl^/ SzJ) [44, 45] or NSG-RIP-DTR [NOD.Cg-Prkdc^scid^Il2rg^tm1Wjl^/ Tg(Ins2-HBEGF) 6832Ugfm/SzJ] mice [46]. Mice were kept in conventional mouse rooms at constant temperature (70–72^°^F) and 12h light/dark cycle with free access to food and water. Day 14 rGBCs were washed with 1X PBS and collected using rubber cell scrapers and transferred into 15-mL conical tubes. rGBC clusters were centrifuged at 1,200 rpm for 7 minutes at 4^°^C and the supernatant was discarded. For transplantation under the kidney capsule, rGBC pellets (5x10^5^-2x10^6^ cells) were mixed with 50 μL whole blood collected from a NSG mouse and allowed to clot. rGBC-clot mixtures were deposited under the left kidney capsule of an anesthetized (isoflurane vaporizer set at 2% with O_2_ flow rate of 1 liter/min) NSG mouse (thirty-four animals received non-reprogrammed GBC and seventeen received rGBC). For transplantation of rGBCs into the mammary fat pad (n = 17 mice), epididymal fat pad (n = 17 mice), and dorsal cutaneous fat (n = 12 mice), the scraped rGBC pellets were suspended in ~40 μL hydrogel (Advanced BioMAtrix, Carlsbad, CA, USA) and injected directly into the fat depots. Between 3–5 independent *in vitro* reprogramming experiments were performed to generate transplantable rGBCs in 3–5 cohorts of mice per transplant site. Post-operative mice were assessed for overall health and wellbeing following standard operating procedures set by OHSU Department of Comparative Medicine. After indicated time points, xenografts from kidneys and fat pads were excised and fixed in 4% paraformaldehyde in 1X PBS for 6–10 hours at 4^°^C. Tissues were washed in 1X PBS and immersed in 30% sucrose in 1X PBS for 18–24 hours at 4^°^C. Fixed tissues were blotted dry, cut into smaller fragments (less than 1 cm^3^ fragments), and embedded in Tissue-tek cryomatrix (Sakura, Tokyo, Japan), frozen in methylbutane/liquid nitrogen, and stored at -80^°^C.

### Immunofluorescence

OCT tissue blocks were cut into 5–8 μm thick cryosections using a Reichert 2800 Frigocut cryostat (Reichert Scientific Instruments, Buffalo, NY, USA) onto Superfrost Plus slides (Fisher Scientific, Pittsburgh, PA, USA) and allowed to dry for 10 minutes. Slides were rinsed three times with 1X PBS, permeabilized with 0.1% Triton X-100 in 1X PBS at room temperature for 10–15 minutes, and washed three times with 1X PBS. Slides were blocked with 5% normal goat serum/normal donkey serum in 1X PBS for at least 30 minutes at room temperature. Slides were incubated with primary antibodies (1:100 dilution v/v) (S2 Table) overnight in a humidified chamber at 4^°^C. Primary antibodies were removed and slides were rinsed three times with 1X PBS. Fluorochrome-conjugated secondary antibodies (goat and donkey; 1:500 dilution v/v) were incubated for 1–2 hours at room temperature. Slides were washed three times with 1X PBS prior to mounting with Fluoromount-G with DAPI (Southern Biotech, Birmingham, AL, USA). Slides were evaluated using a Zeiss Axioskop 2 plus microscope or LSM 700 confocal microscope (Carl Zeiss, Jenna, Germany).

### Statistical analyses

All statistical calculations were performed using Prism 6.0/7.0 (GraphPad Software, Inc., La Jolla, CA, USA), unless otherwise stated. Unpaired t-tests were done to determine statistical significance (α = 0.05). Correlation between two datasets was measured by Pearson correlation coefficient (95% confidence interval).

## Results

### Adult human gallbladder is a rich source of expandable autologous cells for pancreatic endocrine reprogramming

**Fig 1:**
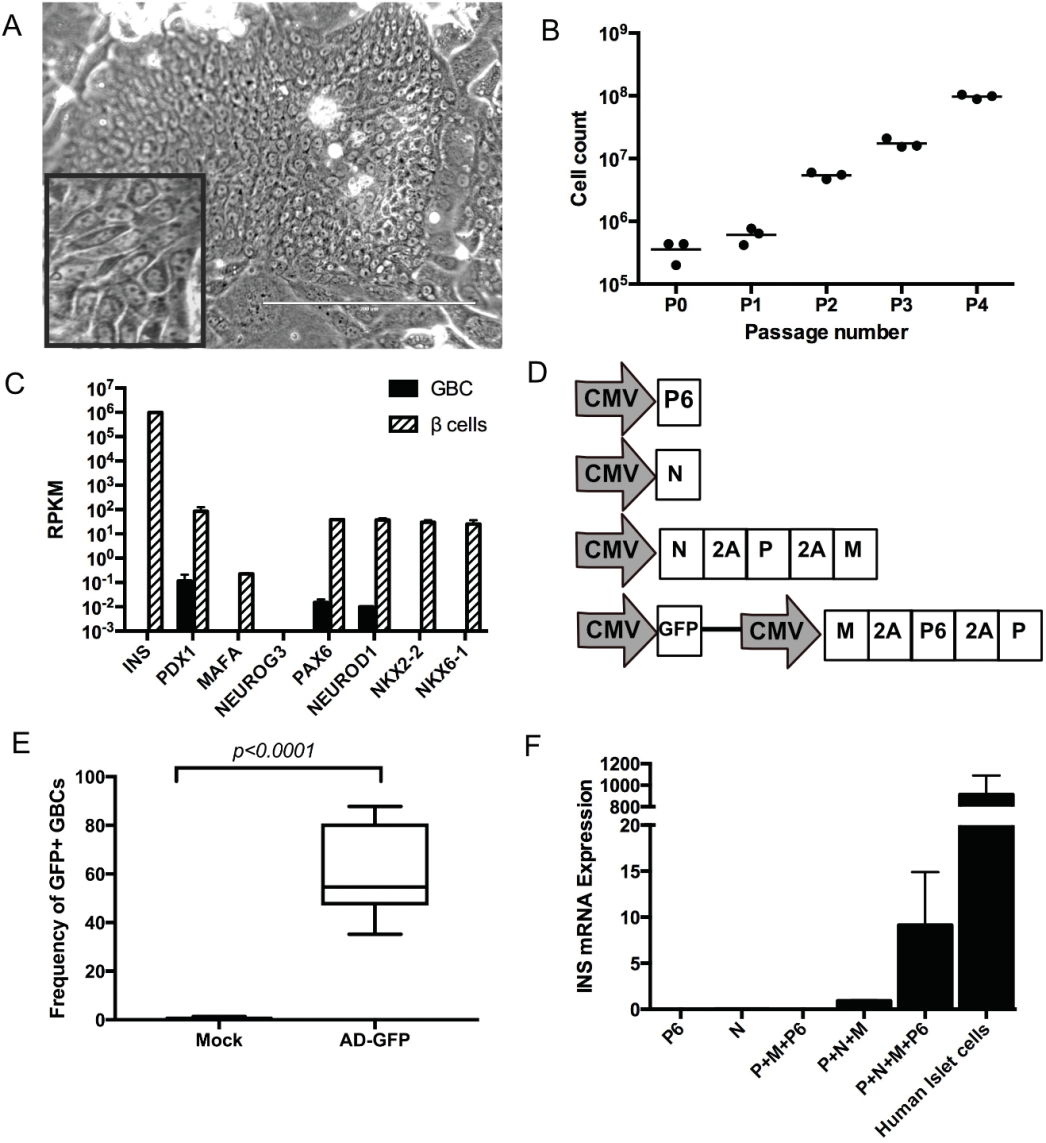
Primary human gallbladder cells (GBCs) *in vitro* culture, expansion, and adenoviral transduction. (A) Two-week old culture of primary human GBCs (scale bar = 200 μm) in a tightly packed colony. Inset shows high nuclear to cytoplasmic ratio. (B) Cell growth of GBCs for the first 4 passages on irradiated LA7 feeder cells. (C) Comparing the gene expression of human primary GBCs with human pancreatic β cells showing differential expression of β cell-associated genes as determined by RNA-seq from duplicate samples. (D) Ad5 vectors encoding for *PAX6* (P6), *NEUROG3* (N), *PDX1* (P), and *MAFA* (M) driven by CMV promoter. The tricistronic vector Ad5-M6P also encoded for green fluorescent protein (GFP). Self-cleaving 2A connects transgenes in multicistronic cassettes. (E) Transduction efficiency of cultured human GBCs to GFP-expressing adenoviral vector (MOI = 500 vg/cell). (F) Insulin mRNA expression in reprogrammed GBCs (rGBCs) five days after transduction with RDAd5 vectors (MOI = 500 vg/cell).

For human gallbladder to be a practical source for β cell replacement, a reliable culture and expansion method needs to be in place. We cultured human GBCs on a subconfluent feeder layer of irradiated LA7-RSPO1 cells and a culture medium modified from murine gallbladder medium [10, 39]. This enabled robust growth and reliable passaging regardless of donor source. Using this protocol, we successfully cultured and serially passaged GBC from over 100 gallbladder and cystic duct specimens ~80% of the time. The addition of cholera toxin (increased cAMP) [47], nicotinamide (increased nicotinamide adenine dinucleotide) [48], N-acetylcysteine (antioxidant) [49], non-essential amino acids, and R-Spondin (enhancement of canonical Wnt signaling) [50] significantly accelerated the expansion of human rGBCs comparable to its murine counterpart, which eventually allowed us to propagate millions of human rGBCs *in vitro*. Cultured GBCs had a high nucleocytoplasmic ratio with prominent nucleoli, a cellular diameter between 7 μm and 12 μm, and grew in tightly packed colonies flanked by the larger irradiated feeder cells (Fig 1A). GBCs could be successfully cultured up to 12 passages allowing for robust expansion *in vitro*. After initial seeding of 5x10^5^ primary cells, GBCs could be expanded to 1x10^8^ cells after 4 passages (Fig 1B) in a span of 4–8 weeks.

To guide our *in* vitro genetic reprogramming and differentiation protocols, we used RNA-sequencing data from both human GBCs and FACS-sorted β cells. The comparative study of the transcriptomes of GBCs and human pancreatic β cells allowed us to ascertain candidate pancreatic endocrine factors for genetic reprogramming. Gene set enrichment analyses of the top 224 differentially expressed genes in human β cells relative to GBCs (log_2_FC>5 and *p*<0.01) indicated that these genes (missing in GBCs) are involved in regulation of β cell development, gene expression, peptidase activity, insulin secretion, synthesis, and processing (S3 Table). The baseline gene expression profile indicated that primary GBCs did not produce insulin and were deficient or expressed very low levels of β cell-associated lineage, differentiation, and identity factors (*PDX1*, *MAFA*, *NEUROG3*, *PAX6*, *NEUROD1*, *NKX2-2*, and *NKX6-1*) (Fig 1C). Previous studies utilized some of the abovementioned transcription factors for the induction of a β-like state in different mouse and human tissues [10, 18–20, 22, 51]. Similar to our previous work with mouse gallbladder [10, 38], we used replication-defective adenovirus serotype 5 (RDAd5) encoding for multiple transcription factors in both monocistronic and multicistronic expression cassettes (Fig 1D and S1 Fig). RDAd5 consistently transduced 60% of GBCs based on GFP expression (Fig 1E). Different combinations of transcription factors were tested, but only the combination of *NEUROG3*/*PDX1*/*MAFA* (*NPM*) and *MAFA*/*PAX6*/*PDX1*/*NEUROG3* (*M6PN*) consistently induced insulin expression (Fig 1F and S1 Fig). Adenoviral transduction of each individual reprogramming factor did not induce expression of insulin or other β cell-associated genes. Transduction of GBCs with monocistronic AD-PDX1 and AD-MAFA (both human codon-optimized) not only failed to induce *INS* but also did not upregulate *NKX6-1*, *NKX2-2*, *NEUROD1*, *PAX6*, endogenous *PDX1*, *MAFA*, *PCSK1 or GLUT2* (S1A, S1B, S1E and S1F Fig). Although AD-NEUROG3 (human codon-optimized) alone did not upregulate the expression of *INS* (Fig 1F), *NKX6-1*, *PAX6*, *or GCK;* it did, however, induce expression of *NKX2-2*, *NEUROD1*, *SST and PCSK1*. (S1C and S1D Fig). In contrast, AD-PAX6 by itself did not induce any pancreatic endocrine factors or hormones (Fig 1F and S1H Fig). Adenoviral-mediated expression of *NKX2-2*, *NKX6-1*, and *PAX4* did not induce *INS*. Together, these experiments show that the combination of 3 factors (PDX1, MAFA and NEUROG3 = PMN) was the minimum requirement for the induction of *INS* expression in human GBCs. Although *PAX6* was not necessary for insulin expression, it significantly enhanced insulin mRNA levels by PMN (Fig 1F and S1 Fig).

### Transduction of M6PN transcription factors induced the pancreatic endocrine program in rGBC

**Fig 2:**
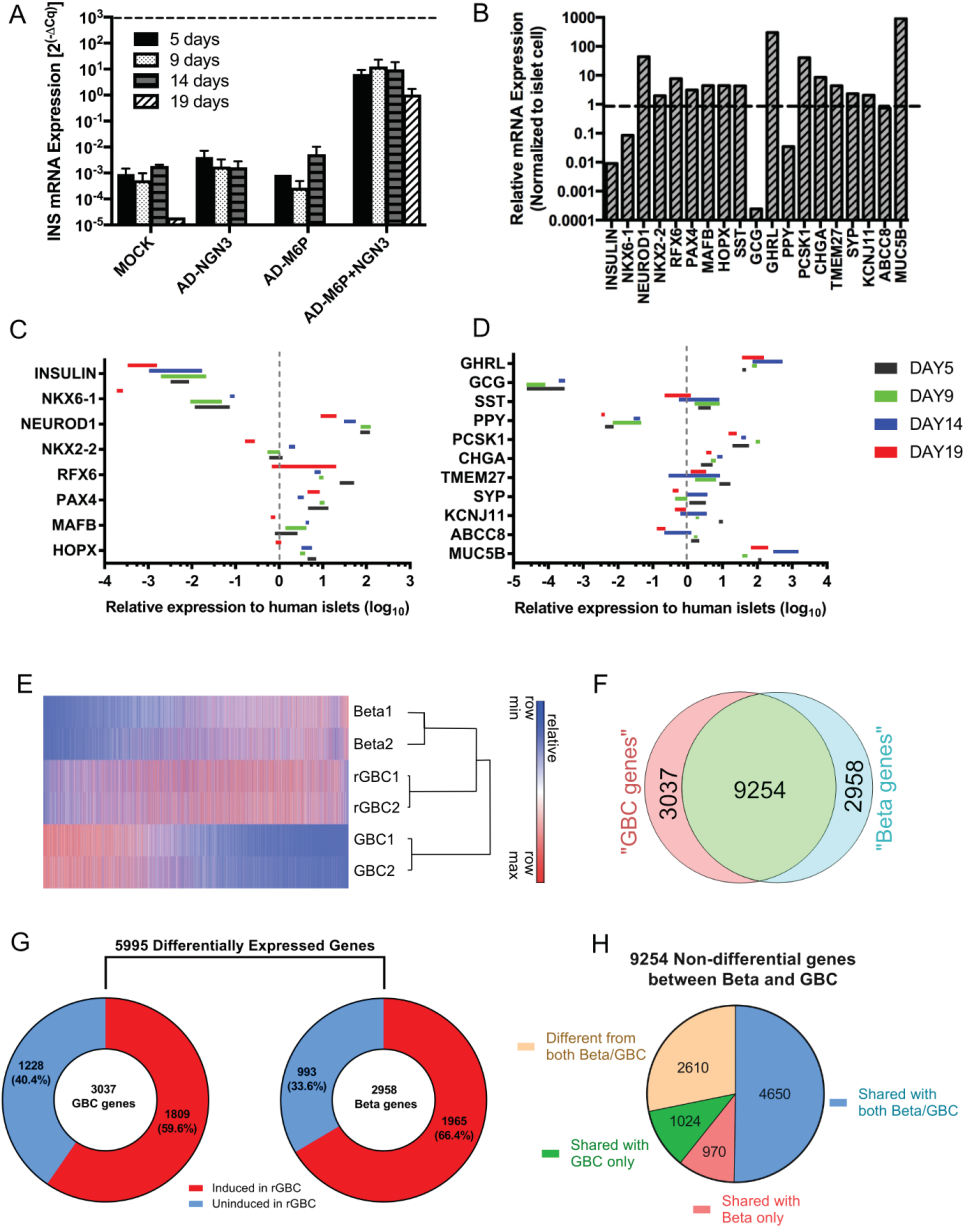
Expression of islet-associated genes in transduced GBCs. (A) Insulin mRNA level expression in GBCs after adenoviral transduction with *NEUROG3* (NGN3), *MAFA* (M), *PAX6* (6), *PDX1* (P) as measured by RT-qPCR. The dashed line denotes insulin mRNA level in human pancreatic islets. (B) Expression levels of pancreatic genes on day 14 in rGBCs as measured by RT-qPCR. *MUC5B* served as a GBC marker. (C-D) M6PN-transduced GBCs were harvested on days 5, 9, 14 and 19 post-transduction. Gene expression levels of pancreatic endocrine factors were measured by RT-qPCR. Relative expression levels were calculated using the formula: [2^(-ΔCq rGBC)]/[2^(-ΔCq human islet)]. The dashed line marks the point where gene expression level is equivalent to human islets. (E) Heat map from clustering of differentially expressed genes in rGBCs in comparison to GBCs and human β cells as determined by RNA-sequencing. (F) Venn diagram depicting non-differential genes (light green) and differentially expressed genes upregulated in GBC ("GBC genes") and β cells ("Beta genes"). (G) Break-down of the frequencies of "GBC genes" and "Beta genes" into induced or uninduced genes in rGBC. (H) Pie chart distribution of the 9254 non-differentially expressed genes in both unreprogrammed GBC and β cells into 4 groups based on global gene expression profile in rGBC.

GBCs transduced with reprogramming factors began to show morphological changes from a monolayer into three-dimensional clusters as early as two days after transduction and cell growth slowed markedly after one week. Cells that were not incorporated into the three-dimensional clusters of rGBC eventually died within two weeks. Induction of insulin expression in reprogrammed GBCs (rGBCs) was evident on day four after viral transduction (S1G Fig). We extended our observation period up to nineteen days to determine the temporal pattern of insulin expression in rGBCs. In M6PN-transduced GBCs, insulin mRNA peaked around nine days and began to decrease slightly after two weeks. Importantly, the combination of all four transcription factors resulted in the highest insulin expression. M6PN-rGBCs had 4 to 5 orders of magnitude greater insulin expression relative to 3-factor (M6P) or 1-factor (N) transduction groups, which also remained consistent over the entire period of observation (Fig 2A). The kinetics of GBC reprogramming was affected not only by the pancreatic endocrine factors introduced but also by the reprogramming media and is consistent with previous work with pancreatic ducts [17]. Pre-treatment of GBCs with DMSO and the addition of retinoic acid, T3, Alk inhibitor (SB431542), and ISX-9 to the reprogramming medium further improved both INS and NKX6-1 levels (S2 Fig). Based on these observations, we performed subsequent reprogramming experiments for a period of 14 days to measure expression levels of β cell-associated factors induced in rGBCs. Quantitative RT-PCR showed that rGBCs upregulated many important β cell-associated genes to levels similar to those seen in intact human islets. Whereas, *INS* and *NKX6-1* were 10–100 times less in rGBCs, respectively (compared to islets), *NEUROD1*, *NKX2-2*, *RFX6*, *PAX4*, *MAFB*, and *HOPX* expression were 5–50 times higher in rGBC compared to pancreatic islets. Other genes related to insulin processing (*PCSK1*) and granule storage/release (*CHGA*, TMEM*27*, *SYP*, *KCNJ11*, *ABCC8*) were likewise strongly upregulated. Among other islet-produced hormones, *GCG* was barely induced while *SST* and *GHRL* were significantly expressed. *PPY* gene expression was around 50 times lower in rGBCs than human islets. While many pancreatic endocrine genes were highly induced, the reprogrammed cells did not fully silence GBC genes. For example, the GBC-related *MUC5B* gene was still highly expressed in rGBCs (Fig 2B). The temporal expression of pancreatic endocrine genes in rGBCs followed the same trajectory as *INS*—peaking between day 9 and 14 and declining afterwards (Fig 2C and 2D). With the exception of INS, NKX6-1, and PPY, all other pancreatic endocrine-associated genes (e.g. *NEUROD1*, *NKX2-2*, *RFX6*, *PAX4*, *MAFB*, *HOPX*, *SST*, *GHRL*, *PCSK1*, *CHGA*, *TMEM27*, *SYP*, *KCNJ11*, *ABCC8*) had greater expression levels than whole human islets (Fig 2B–2D). Overall, insulin-producing rGBCs showed a β-like expression profile with additional expression of δ, ε, and γ endocrine genes, but also retained a partial biliary character. To further evaluate the global effects of genetic reprogramming on human GBCs, we compared the complete transcriptomes of unreprogrammed GBCs and human β cells to rGBCs using RNA-sequencing. The heat map illustrated that rGBCs significantly upregulated genes that are associated with β cell state while downregulating genes that are more characteristic of parental GBCs (Fig 2E). The total number of expressed genes detectable in both human β cell and GBC was 15,249 (RPKM>1), with 9,254 (60.7%) genes not differentially expressed [ABS(log_2_FC)<1 and/or *p*>0.01)]. There were 5,995 differentially expressed genes ([ABS(log_2_FC>1 and *p*<0.01), whereby 3,037 (19.9%) genes were more highly expressed in GBCs (“GBC genes”) while 2,958 (19.4%) were upregulated in human β cells (“Beta genes”) (Fig 2F). Of these 2,958 “Beta genes”, 1,965 (66.43%) were induced in rGBCs (Fig 2G). Gene set enrichment analysis of the 151 most differentially expressed of these “Beta genes” in rGBCs (log_2_FC>5 and *p*<0.01) demonstrated that rGBCs upregulated the molecular signatures involved in the regulation of (a) β cell development, (b) gene expression in β cells, (c) MODY (Maturity onset diabetes of the young), (d) and Type 1 Diabetes Mellitus (S4 Table). However, the analysis also revealed that 993 “Beta genes” remained uninduced in the rGBC population. Gene set analysis showed that these uninduced “Beta genes” in rGBC are involved in generic transcription activity, extracellular matrix proteins, plasma membrane proteins and nervous system development (S5 Table). Notably, *ZNF* family of genes encoding for Krueppel C2H2-type zinc-finger family of proteins were well represented (35 out of 44 genes) among the transcription regulators (Reactome_Generic Transcription pathway) which were differentially expressed in human β cells but not in rGBCs. In addition, 1,809 out of 3,037 (59.6%) “GBC genes” remained expressed in rGBCs suggesting a residual biliary signature (Fig 2G and S6 Table). Interestingly, 2,610 genes (28.2%) were unique to rGBC and differentially expressed compared to both unreprogrammed GBC and human β cells (Fig 2H).

**Fig 3:**
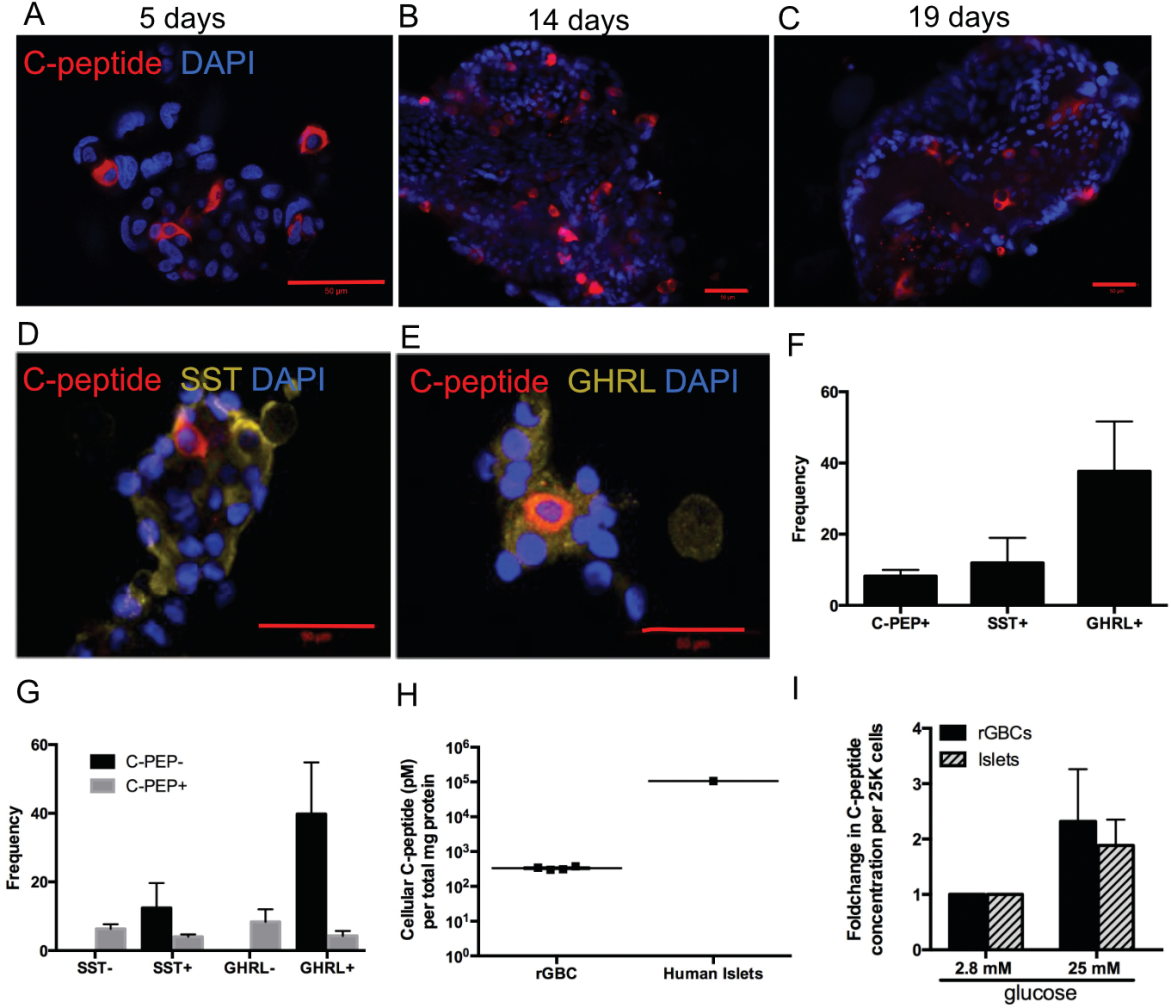
Detection of pancreatic endocrine proteins in transduced GBCs. Immunofluorescent staining of human C-peptide (A-E), SST (D), and GHRL (E) in transduced GBCs (scale bar = 50 μm). Frequencies of single (F) and dual (G) immunofluorescent staining for C-peptide, SST and GHRL in rGBCs. Intracellular (H) and stimulated (I) C-peptide protein levels in rGBCs relative to human islets.

In addition to gene expression analysis, reprogramming was also assessed at the protein level. Consistent with the gene expression data, insulin protein production (as measured by C-peptide) was found on day five (Fig 3A), persisting at two weeks (Fig 3B) and lasting for at least nineteen days (Fig 3C) after viral transduction. Human C-peptide in rGBCs was localized in the cytoplasm and had a characteristic punctate appearance (S3A Fig). Some of the C-peptide+ rGBC also co-expressed NKX6-1, NKX2-2, NEUROD1, and PP (S3B–S3G Fig). Staining patterns of NKX6-1, NKX2-2, and NEUROD1 were indicative of nuclear and/or cytoplasmic localization. Consistent with gene expression experiments, GCG was not detected by immunostaining. Reprogrammed cells were heterogeneous in their hormone expression patterns. 7.11±4.18% stained only for C-peptide, 11.9±15.7% only for SST and 37.67±24.3% GHRL (Fig 3D–3F). In addition, many polyhormonal cells were also present. There were also C-peptide-positive rGBCs which concomitantly stained for SST (4.01%) and GHRL (4.28%) (Fig 3G). Moreover, vector-encoded transgene as indicated by GFP showed continued expression (S3H Fig). In aggregate, these data show that our reprogramming protocol induced not only a β-like state but also limited δ and ε-like states.

To further determine functional characteristics of β cells, rGBCs were tested for their ability to release insulin in response to glucose. The bulk rGBC population had an intracellular C-peptide content 2 orders of magnitude lower than intact human pancreatic islets (Fig 3H), taking into account that approximately 10% of total rGBC were C-peptide positive (Fig 3F). The effective intracellular amount was calculated to be 10% of that produced in intact islets. Moreover, similar to intact human islets, rGBCs exhibited good response to glucose stimulation *in vitro* (Fig 3I).

### Enrichment for insulin-producing rGBCs by FACS using a pan-islet endocrine cell surface-reactive monoclonal antibody

**Fig 4:**
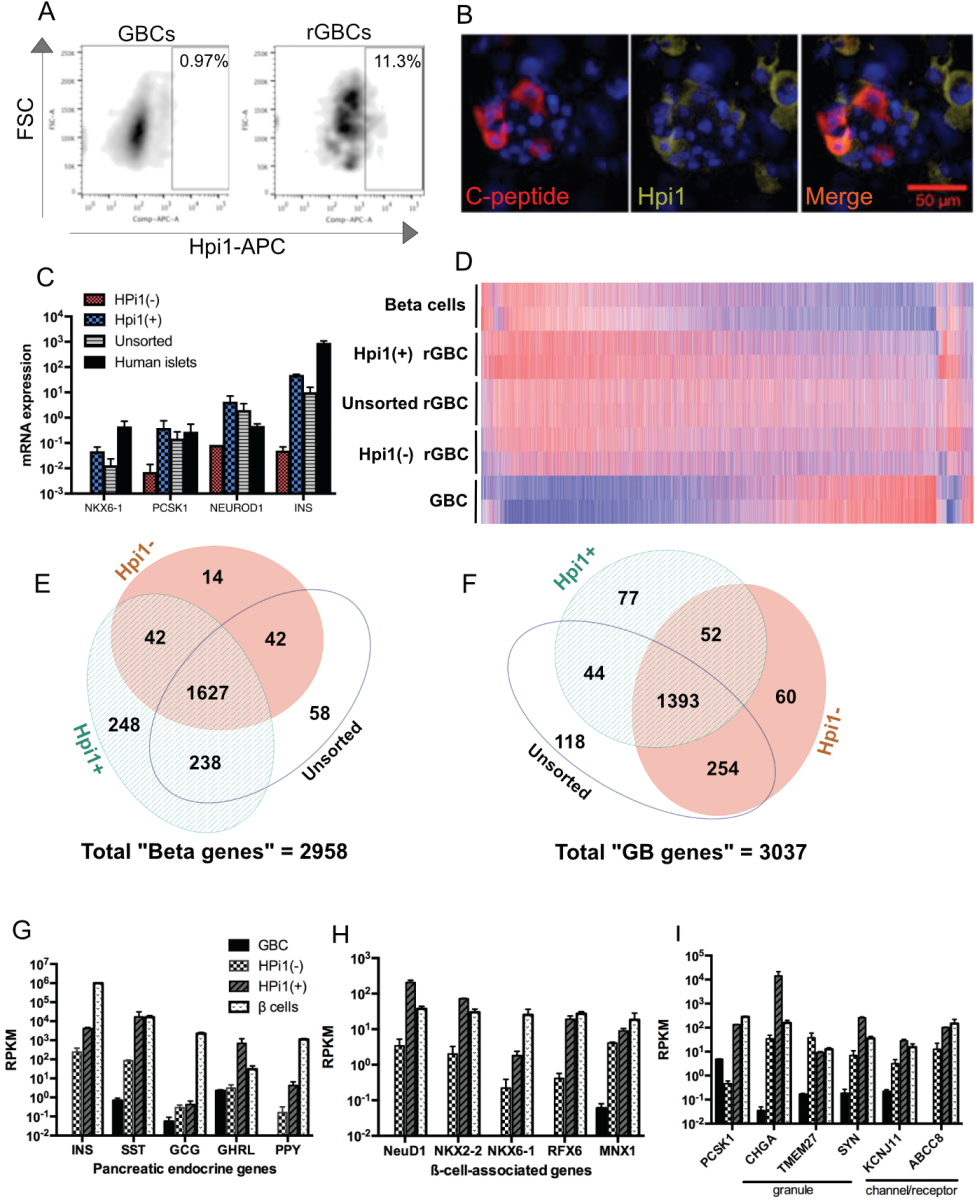
The human pan-endocrine antibody Hpi1 enriched for β-like cells. (A) Flow cytometry dot plots showing frequencies of Hpi1+ cells in both GBCs and rGBCs. (B) Immunofluorescent images of C-peptide co-localizing with the antigen for Hpi1. (scale bar = 50 μm) (C) mRNA expression levels of selected β-associated genes in Hpi1-based FACS-sorted rGBCs showing enrichment for *NKX6-1*, *PCSK1*, *NEUROD1*, *and INS* in Hpi1+ rGBCs by RT-qPCR. (D) Heat map of differentially expressed genes in sorted rGBCs by RNA-sequencing. Venn diagrams showing overlapping and non-overlapping "Beta genes" (E) and "GB genes" (F) upregulated in Hpi1-/+ and unsorted rGBC as measured by RNA-seq. Differential expression of pancreatic endocrine genes (G), β cell-associated transcription factors (H), and genes involved in the regulation of insulin secretion (I).

The C-peptide+ rGBCs generated by our protocol also included rGBCs positive for other islet hormones (Figs 2B–2D, 3D–3G and S3 Fig). To separate and enrich for *INS*+ rGBC, we tested the human islets cell surface markers (Hpi1 and Hpi2) previously used to specifically isolate primary human islet cells by FACS [40, 52]. Using Hpi1, we were able to enrich insulin-positive rGBCs by flow cytometry. Genetic reprogramming of GBC using M6PN resulted in the upregulation of Hpi1-reactive surface antigen by a factor of 11.6 (Fig 4A). C-peptide-positive rGBCs were exclusively Hpi1**+** by immunocytochemistry (Fig 4B). FACS separation based on Hpi1 staining also resulted in the enrichment β-cell specific genes. Hpi1**+** rGBCs had marked increases in the mRNA expression of *INS* (4.8-fold increase) (*p* = 0.024), *NKX6-1* (3.6-fold increase), *NEUROD1* (2.12-fold increase), and *PCSK1* (2.6-fold increase) over that of the unsorted bulk rGBC population. As expected the gene expression differences were markedly higher when Hpi1+ cells were compared to Hpi–cells. NKX6.1 was increased by 4.7x10^3^-fold, *PCSK1 by* 55-fold and NEUROD1 by 50-fold. Moreover, *INS* gene expression was >1,000 higher in Hpi1**+** than Hpi1– rGBCs (*p* = 0.0023) (Fig 4C). In contrast, the pan-islet surface antibody Hpi2 did not enrich for *INS* or any other pancreatic endocrine genes (S4A and S4B Fig).

Further examination of the transcriptomes of the different rGBC subpopulations showed that global gene expression signature of Hpi1+ rGBCs clustered most closely to human pancreatic β cells followed by unsorted rGBCs and then by Hpi1- rGBCs. All 3 subsets of rGBCs have gene expression profiles distinct from the unreprogrammed GBCs (Fig 4D). Genes that are poorly expressed in unreprogrammed GBCs are relatively highly expressed in β cells and rGBC populations, particularly in Hpi1**+** rGBCs. On the corollary, genes that are highly expressed in unreprogrammed GBCs are relatively downregulated in rGBCs and β cells. Of the 2,958 “Beta genes” or genes differentially upregulated in human β cells, 2,155 (72.8%) genes are also upregulated in Hpi1**+** rGBC versus the 1,725 (58.3%) in Hpi1***-*** rGBC and the 1,965 (66.4%) in unsorted rGBC. Notably, Hpi1+ rGBC expressed 248 (8.4%) unique “Beta genes” as opposed to only 14 (0.47%) genes in Hpi1- rGBC and 58 (2.0%) in unsorted rGBC (Fig 4E). Gene set enrichment analysis of the 248 “Beta genes” exclusively upregulated in Hpi1+ but not in Hpi1- and unsorted rGBC, revealed gene categories involved in voltage-gated potassium channels, synaptogenesis, nervous system development and differentiation (S7 Table). Overall there were 1,949 (65.9%) “Beta genes” similarly upregulated in at least 2 groups of Hpi1**+**/- and/or unsorted rGBCs (Fig 4E). Moreover, Hpi1**+** rGBC retained the lowest proportion of “GB genes” at 1,566/3,037 (51.6%) compared to Hpi1- (1,759/3,037 or 57.9%) and unsorted rGBC (1,809/3,037 or 59.6%) (Fig 4F). Therefore, FACS enrichment markedly improved the overall β-like molecular signature or rGBCs as evidenced of Hpi1**+** rGBC being more enriched in “Beta genes” and less biliary (“GB genes”) character compared to Hpi1- or unsorted rGBC populations. Gene set investigation of “GB genes” still highly expressed in Hpi1+ rGBC indicates roles in the extracellular matrix, integral proteins in the plasma membrane, receptor interactions, calcium ion-binding, and polysaccharide biosynthesis and degradation (S8 Table).

Given that Hpi1 is a pan-islet endocrine surface marker, Hpi1**+** FACS-sorted rGBCs enriched not just for *INS*, but also for other islet endocrine genes such as SST, *GHRL*, and *PPY* with the exception of *GCG* (Fig 4G). This suggests that the antigen for Hpi1 does not exclusively associate with insulin-producing cells but with pancreatic endocrine cells in general. More importantly, the expression of hallmark β genes such as *NEUROD1*, *NKX2-2*, *NKX6-1*, *RFX6*, and *MNX1* that are associated with β cell fate, identity, function, and maintenance were thoroughly enriched in Hpi1+ rGBCs (Fig 4H). Furthermore, Hpi1+ rGBCs had greater expression of genes related to insulin processing (*PCSK1*), packaging (*CHGA*, *SYN*), and response to glucose (*KCNJ11*, *ABCC8*) relative to Hpi1- rGBC, except for *TMEM27* (Fig 4I).

### microRNA profiling of reprogrammed human gallbladder cells

**Fig 5:**
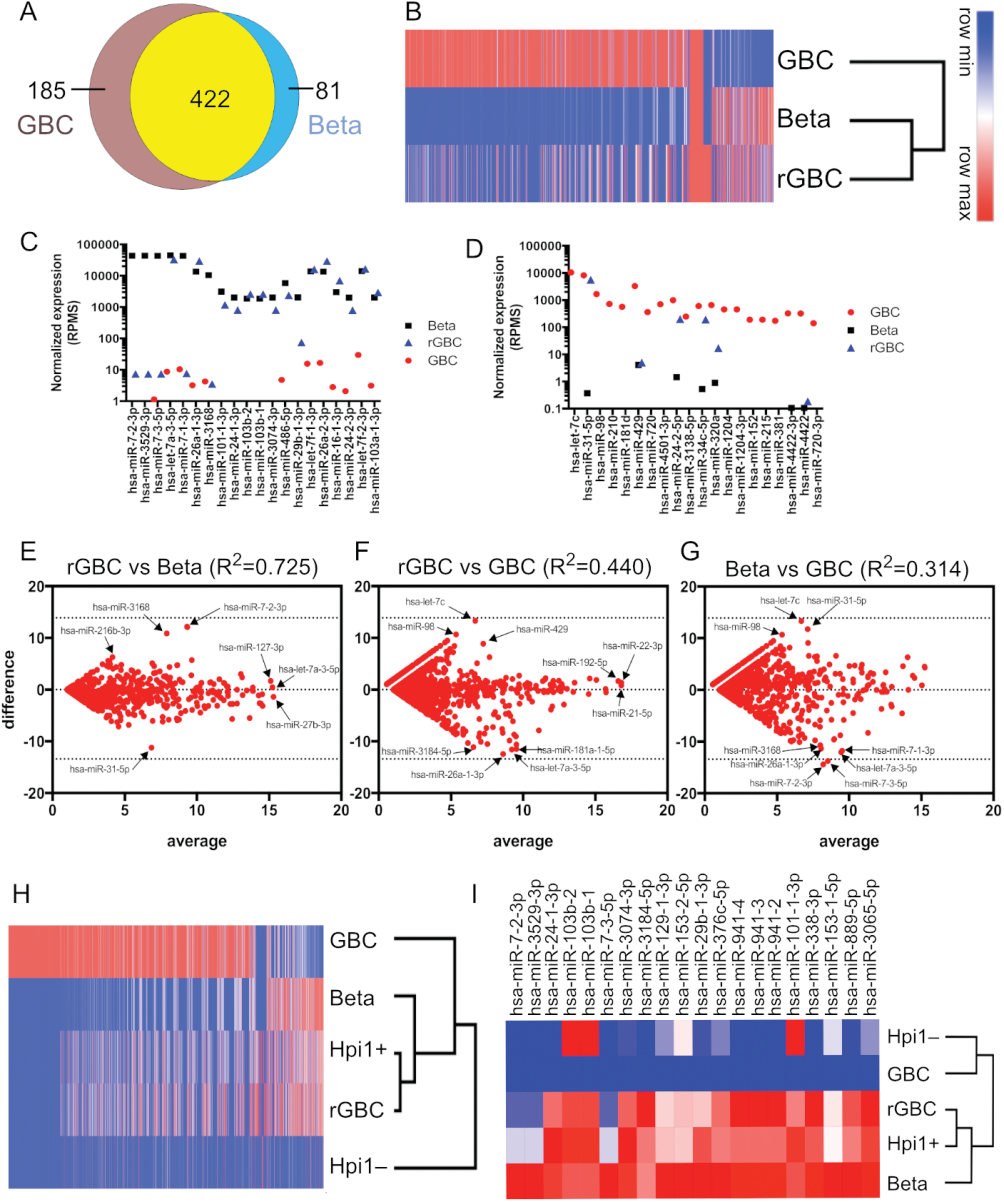
Determination of microRNA expression in rGBC relative to human pancreatic β cells and primary GBC by Illumina sequencing. (A) Venn diagram of microRNAs differentially expressed in human pancreatic β cells (blue) or primary GBCs (brown) or no difference (yellow). (B) Heat map and dendogram of microRNAs in β cells, primary GBC, and rGBC. (C,D) Top twenty differentially expressed microRNAs enriched in β cells (C) and GBC (D). (E-G) Bland-Altman plots comparing the microRNA populations in β cells, primary GBC, and rGBC. Y-axes correspond to expression differences [log_2_(rGBC–β, GBC–rGBC, GBC–β, respectively)] and the X-axes correspond to average value of each microRNA in each paired cell types. Selected microRNAs are annotated to illustrate differential expression among the three cell types. (H) Hierarchical cluster analysis of microRNAs in Hpi1 subpopulations relative to unsorted rGBC, primary GBC, and β cells. (I) Heat map distribution of the twenty most differentially expressed microRNAs enriched in β cells and downregulated or absent in primary GBC across clustered cell types.

The development, cellular identity, function, and physiologic state of pancreatic β cells are governed by interactions or network of both coding and non-coding RNAs [53, 54]. Above, we demonstrated that rGBCs displayed a similar β cell mRNA expression profile. Given that the reprogramming strategy used was based on ectopic gene expression of pancreatic endocrine transcription factors, we sought to determine whether this reprogramming also resulted in a β cell non-coding RNAs profile. There were 1,896 high confidence (based on miRbase v21) [55] mature microRNAs enumerated in our small RNA library sequencing, 894 microRNAs present in both human β cells and primary GBCs, 63 exclusively β cells, 705 exclusively in primary GBCs, and 235 microRNAs were absent in both cell types. Of the 894 microRNAs detectable (RPM>1) in both GBC and β cells, 81 were more highly expressed in β cells (RPM>5 and FC>10), and 185 enriched in primary GBC (RPM>5 and FC>10). In addition, there were 422 that were not differentially expressed (FC<10 and/or RPM<5 in both cell types) (Fig 5A). Unbiased hierarchical clustering revealed that the global microRNA contents of rGBC had a closer relationship with β cells than with primary GBCs (Fig 5B). Of the 81 β cell-specific microRNAs, 54 (60.1%) were upregulated in rGBCs while only 14 (7.57%) out of the 185 GBC microRNAs remained expressed in rGBCs. Moreover, fifteen of the twenty most highly expressed β microRNAs showed robust expression in rGBC compared to GBC (Fig 5C). In contrast, the top twenty GBC microRNAs were poorly represented (four out of twenty) in rGBC (Fig 5D). Taking into account the overall microRNA expression profiles, there was even stronger correlation between rGBC and β cells (R^2^ = 0.725) compared to rGBC and primary GBC (R^2^ = 0.440) (Fig 5E). This finding provides further evidence that rGBCs had a microRNA expression levels highly similar to primary human β cells.

MicroRNAs that were significantly upregulated in both rGBC and β cells are listed on Table 1 and selected microRNAs were plotted relative to their expression differences in GBC, rGBC, and β cells (Fig 5E and 5F). Notably, microRNAs hsa-miR-127-5p, hsa-miR-370 and hsa-miR-376 had been shown to be highly and specifically expressed in islets of developing and adult human pancreas [53, 54]. β-associated microRNAs that were either absent or downregulated in rGBC are listed on Table 1. The abundant β microRNAs lacking in rGBC included hsa-miR-3168, –7-1-3p, –7-2-3p, –7-3-5p,[43, 53] and –216b-3p (Fig 5E and 5G). If these microRNAs have significant roles in β cell fate and function, they represent potential targets for the improvement of GBC reprogramming.

**Table 1:** List of β cell-enriched and GBC-enriched microRNAs that were differentially represented in rGBC.

β microRNAs upregulated in rGBC	β microRNAs downregulated or absent in rGBC	GBC microRNAs upregulated in rGBC	GBC microRNAs downregulated or absent in rGBC
hsa-miR-3184-3p, -5p	hsa-miR-216b-3p	hsa-miR-135b-3p, -5p	hsa-let-7a-2-5p, -7c
hsa-let7a-1-3p,-3-3p,-5p	hsa-miR-670-3p	hsa-miR-31-3p, -5p	hsa-miR-1180, -1183-5p
hsa-miR-103a-1-3p	hsa-miR-1179	hsa-miR-34b-5p	hsa-miR-1234-5p
hsa-miR-103b-1, -2	hsa-miR-3168	hsa-miR-34c-5p	hsa-miR-1273a-5p
hsa-miR-153-1-5p, -2-5p	hsa-miR-129-1-5p	hsa-miR-452-3p, -5p	hsa-miR-1280, -1287
hsa-miR-24-1-3p, -2-3p	hsa-miR-7-2-3p, -5p	hsa-miR-181a-1-3p	hsa-miR-128-1
hsa-miR-3065-3p, -5p	hsa-miR-3529-3p, -5p	hsa-miR-196b-5p	hsa-miR-1296
hsa-miR-338-3p, -5p	hsa-miR-7-3-3p, -5p	hsa-miR-222-5p	hsa-miR-1301, -1303-5p
hsa-miR-374b-5p	hsa-miR-7-1-3p	hsa-miR-224-5p	hsa-miR-210, -215
hsa-miR-374c-3p	hsa-miR-217	hsa-miR-24-2-5p	hsa-miR-219-1-3p
hsa-miR-376a-1-3p, -2-3p		hsa-miR-584-5p	hsa-miR-1468
hsa-miR-376b-5p			hsa-miR-152
hsa-miR-376c-5p			hsa-miR-181d
hsa-miR-423-3p, -5p			hsa-miR-190a
hsa-miR-941-2, -3, -4			hsa-miR-1973-5p
hsa-miR-101-1-3p			hsa-miR-134, -137-5p
hsa-miR-1180-5p			hsa-miR-2276
hsa-miR-1185-2-3p			hsa-miR-3138-5p
hsa-miR-127-5p			hsa-miR-3154-5p
hsa-miR-129-1-3p			hsa-miR-320a-5p, -328
hsa-miR-1301-5p			hsa-miR-3615-5p
hsa-miR-16-1-3p			hsa-miR-3657-5p
hsa-miR-19b-1-3p			hsa-miR-3676-3p, -5p
hsa-miR-197-5p			hsa-miR-376c
hsa-miR-26a-1-3p			hsa-miR-381, -384-5p
hsa-miR-29b-1-3p			hsa-miR-3907-5p
hsa-miR-301a-5p			hsa-miR-410
hsa-miR-3074-3p			hsa-miR-4257-5p
hsa-miR-328-5p			hsa-miR-4268-5p
hsa-miR-370-5p			hsa-miR-4290-5p
hsa-miR-381-5p			hsa-miR-4322-5p
hsa-miR-410-5p			hsa-miR-4419b-5p
hsa-miR-431-3p, -432-5p			hsa-miR-4432-5p
hsa-miR-486-5p,-487b-5p			hsa-miR-4419b-5p
hsa-miR-494-5p, -495-5p			hsa-miR-4432-5p
hsa-miR-625-3p			hsa-miR-4449-5p
hsa-miR-668-5p			hsa-miR-4484-5p
hsa-miR-758-5p			hsa-miR-4485-5p
hsa-miR-889-5p			hsa-miR-4490-5p
			hsa-miR-4734-5p
			hsa-miR-4741-5p
			hsa-miR-487b
			hsa-miR-494
			hsa-miR-504
			hsa-miR-513b
			hsa-miR-548e
			hsa-miR-552
			hsa-miR-5695-5p
			hsa-miR-575-5p
			hsa-miR-598
			hsa-miR-636-5p
			hsa-miR-644b-3p,-646-5p
			hsa-miR-651
			hsa-miR-664-3p,-5p
			hsa-miR-720,-3p
			hsa-miR-760-5p
			hsa-miR-874
			hsa-miR-889
			hsa-miR-98

Although the overall microRNA expression profile of rGBC looked more similar to β cells, there were several GBC microRNAs that were still upregulated in rGBC (Table 1). Scatterplot correlation analysis revealed hsa-miR-31-5p as one of the most differentially expressed microRNA in rGBC and primary GBC that is low to absent in β cells (Fig 5E and 5G and Table 1). These microRNAs are possible targets for suppression to improve the β cell fate conversion of GBC in the future.

We have shown in this study that the surface antibody Hpi1 was effective in enriching for C-peptide+ rGBC. The transcriptome profile of Hpi1+ rGBC was more β-like than unsorted rGBC, while the overall microRNA expression profiles of Hpi1+rGBC and unsorted rGBC were equally very similar to β cells (R^2^ = 0.767 versus R^2^ = 0.766, respectively) (S5A Fig). Concordant with the RNAseq data, the microRNA profile of Hpi1– rGBC was significantly different from Hpi1+ rGBC, unsorted rGBC, and β cells (R^2^:[0.135–0.5844]) (Fig 5H). Furthermore, fourteen of the twenty most differentially abundant β microRNAs were highly expressed in Hpi1+ rGBC, which was greater than the percentages in unsorted rGBC and Hpi1- rGBC (Fig 5I). On the other hand, none of the twenty highest GBC microRNAs were expressed in rGBC subpopulations (both unsorted and Hpi1+/- cells) (S5B Fig). Unsorted rGBC and Hpi1+ rGBC both overexpress hsa-miR-31-5p that was enriched in primary GBC, suggesting that this microRNA is part of the extrahepatic biliary signature. MicroRNAs hsa-miR-3168 and hsa-miR-7-1-3p were both deficient in Hpi1+ and unsorted rGBC (S5C and S5D Fig) and are potentially useful to improve reprogramming. The MicroRNA profile of Hpi1- rGBC correlated poorly with β cells and lacked more β microRNAs relative to unsorted/Hpi1+ rGBC (S5E Fig). Overall, Hpi1+ rGBC efficiently embodied the transcriptomic and microRNA expression profiles of real β cells.

### Transplantation of *ex vivo* reprogrammed human gallbladder cells into immunodeficient mouse

**Fig 6:**
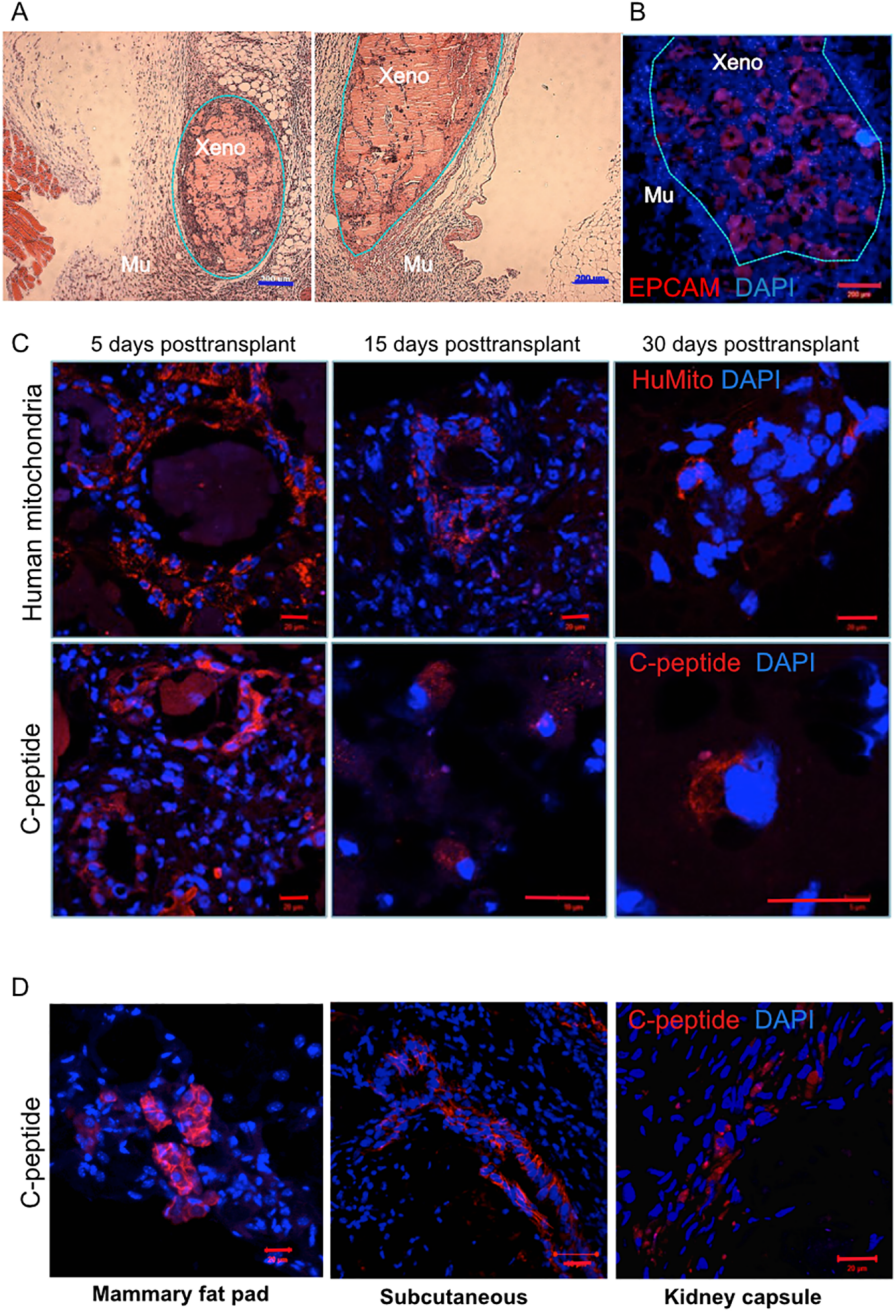
Transplantation of rGBCs into NSG mouse. (A) H&E sections of engrafted rGBCs (Xeno) into the epididymal fat (n = 17) surrounded by mouse tissue (Mu) (scale bar = 200 μm). (B) Immunofluorescent staining of human EPCAM marking the location of rGBCs in the epididymal fat (scale bar = 200 μm). (C) Human mitochondria and C-peptide immunofluorescence of rGBC grafts at three time points after transplantation into the epididymal fat. (D) C-peptide immunofluorescence of rGBC clusters 2 weeks after transplant into mammary fat pad (left) (n = 17), upper back subcutaneous area (middle) (n = 12), and kidney capsule (right) (n = 17) (scale bar = 20 μm).

*INS*+ rGBC survived on average *in vitro* for three to four weeks. We tested whether xenotransplantation would improve survival, maturation, and functionality. 5x10^5^ to 2x10^6^ cells from 14-day old rGBC clusters were transplanted into several different locations including the kidney capsule, mammary fat pad, interscapular white adipose depot, and epididymal fat pad (EFP). Hematoxylin and eosin staining of 2-week old human rGBC xenografts in the EFP were well-circumscribed with hypocellular central areas (Fig 6A) indicating that surviving rGBC congregated in peripheral areas. rGBC xenografts stained positive for both human EPCAM and mitochondria, which were used to assess the extent of the engraftment at 3 different time points up to 30 days (Fig 6B and 6C). C-peptide immunostaining was consistently evident early on day 5 post-transplant and declined over time with only rare C-peptide+ cells remaining after one month in the EFP (Fig 6C). Other sites where rGBC engrafted include the mammary fat pad, subcutaneous fat depot at the upper back, and kidney capsule (Fig 6D). In addition to insulin expressed, rGBC grafts were positive for SST and NEUROD1 (S6A and S6B Fig). Irrespective of the transplantation site and evidence of infiltrating host CD31+ endothelial cells (S6C Fig), all rGBC grafts survived less than 4 weeks, with the highest frequency of human C-peptide+ rGBC seen in the first five days. The expression of adenovector-encoded transgenes as measured by GFP (Ad-M6P) persisted *in vivo* for at least 2 weeks post-transplant or 4 weeks post-transduction (S6C Fig). Twice to thrice weekly testing of blood plasma from NSG mouse recipients yielded no detectable circulating C-peptide at any given time point.

In order to promote long-term engraftment of rGBC, we tested the addition of endothelial (HUVEC) and human mesenchymal stem cells (MSC) into the *in vitro* reprogramming of human GBC starting on reprogramming day 10 (or 10 days post-transduction). Incorporating HUVEC and MSC with cells from various tissues to form organotypic structures *in vitro* had been utilized for successful engraftment [56–58]. We mixed a ratio of 1x10^6^ 10-day old rGBC with 5x10^5^ HUVEC and 2x10^5^ MSC. Between 24 and 48 hours, this heterotypic monolayer culture started to condense into a tight structure which became more tissue-like in consistency after five days (S6D Fig). Prior to transplantation, we checked the relative expression profile and ability to secrete C-peptide in response to glucose stimulation by rGBC:HUVEC:MSC aggregates (S6E and S6F Fig). Visible grafts were observed after 2 weeks and immunohistochemical staining revealed that rGBC (C-peptide) and HUVEC cells (huCD31) were rare to absent, while MSC (huCD44) was abundant at that time point (S3D–S3F Fig). Hence, mesenchyme-driven condensation of rGBC *in vitro* did not enhance the engraftment of rGBC in immunodeficient mice compared to direct transplantation of rGBC clusters.

## Discussion

Our study is the first report of a robust methodology for expansion culture and genetic reprogramming of primary human gallbladder cells from more than a hundred unrelated donors. Previous attempts at *in vitro* cultures of human gallbladder tissues were hampered by inefficient plating, poor passagability, limited cell yield, and fibroblast contamination [59–62]. Our reliably scalable *in vitro* expansion of GBCs is a vast improvement from previous methods as evidenced by a greater than 80% plating and multiple passaging efficiency based on more than one hundred distinct gallbladder specimens, the majority of which were smaller than 5 cm^3^ in dimension. This new method provided us with millions of primary human GBCs which we genetically reprogrammed with key pancreatic transcription factors.

Our rGBC highly recapitulated the β-like cell state not only in its strong insulin production, response to glucose challenge, and global gene expression signature but also in the overall microRNA expression profile. These are indications that ectopic expression of four transgenes (*i*.*e*. *M6PN*) can drastically remodel both the coding and non-coding genetic landscape of GBC to assume a different cellular state (*i*.*e*. β-like cell). As we and others had previously reported, the minimal requirement for *PNM* to reprogram mouse gallbladder [10], mouse pancreatic acinar/ductal cells [17, 20, 22], and mouse liver cells [19] parallels that of reprogramming human GBC, further suggesting that a ductal phenotype shared by GBC [63–65] with epithelial cells in the liver and pancreas is conducive to genetic reprogramming using *PNM*. The high reprogramming potential of endoderm-derived ductal cells as evidenced by this report and others [10, 17, 19, 20, 22] is correlated to their epigenetic proximity (biliary tree and ventral pancreas) [34]. Majority of these studies using the same three reprogramming factors utilized rodent cells/organs as targets for reprogramming. Without taking into account for the inherent differences in species or cellular microenvironment (e.g. *in vitro vs*. *in vivo*, primary *vs*. transformed lines, transdifferentiation protocol for *in* vitro studies, timing of assays), the comparative reprogramming efficiencies of other adult endoderm derivatives like rat and mouse exocrine cells [20, 22], gastric antral cells [33], and Sox9+ cells in the liver [18, 19] were better than human rGBCs. However, contrary to liver, stomach, and pancreas reprogramming, the gallbladder has the advantages of easy access for surgical removal without any dire health sequelae and from a practical perspective—scalable *ex vivo* expansion [10].

Among major β cell transcription factors lacking in human GBC, only the addition of *PAX6* to *PNM*—but not *NEUROD1*, *NKX6-1*, *NKX2-2*—enhanced reprogramming. *PAX6* is one of the major regulator of pancreatic endocrine transcription and determinant of normal islet development [66] and was not induced in GBC after *PNM* transduction. Although PAX6 was not required for GBC reprogramming, its addition to PNM factors clearly improved the induction of insulin and key β-associated genes, especially *NKX6-1*, *NEUROD1*, *NKX2-2*, and *RFX6*. Interestingly, several of these key endocrine genes were induced at higher levels compared to whole human islets. Many of these activated genes are expressed downstream of the reprogramming factors [67]. These adenovirally encoded reprogramming factors M6PN were constitutively overexpressed in rGBCs under the potent CMV promoter, thus, potentially induced downstream genes at significantly higher levels than found in human islets. Consequently, it is still not known the most optimal stoichiometry (or even factor combinations) and actual expression levels of reprogramming factors needed for human gallbladder to completely and efficiently convert to mature and long-lived β-like cells. Definitely, forced overexpression of master reprogramming factors will most likely lead to unregulated and supraphysiologic levels of target downstream genes as observed in this study. Unlike that seen in mouse gallbladder reprogramming [10], *GCG* was not upregulated in human rGBC. A previous study had implicated PAX6 with the activation of the *GCG* promoter [17], which we did not observe in this study, pointing to other binding partners neither available nor induced in human rGBC. It’s been posited that the extrahepatic biliary tree (GBC included) and the ventral pancreas arose from the same SOX17+ progenitor cell [34]. Interestingly, studies performed in rat islets showed that the ventral pancreas to be glucagon-poor while the dorsal pancreas to be glucagon-rich [68–70]. Therefore, if rGBC reprogramming was biased towards a “ventral pancreatic endocrine” state, then this could point to why rGBCs had absent glucagon expression.

Compared to a previous study in primary human pancreatic CD133+ ductal cells (huP133+) [17] the transduction of the same four transcription factors M6PN in human GBCs induced greater insulin gene induction (0.01 rGBC *vs*.0.002 huP133+ relative to human islets). The insulin induction was even higher in Hpi1+ rGBC (>0.05). Both rGBCs and reprogrammed huP133+ had comparable insulin secretion response to high glucose. Overall expression of β cell-associated genes in rGBCs were either greater or comparable to reprogrammed huP133+ [17]. In comparison to primary mouse rGBCs, human rGBCs yielded relatively better *in vitro* reprogramming efficiency after transduction with M6PN factors [38]. However, both reprogrammed huP133+ and mouse rGBCs were able to mature and survive longer *in vivo* [17, 38].

Given the highly similar transcriptomes and microRNAomes of rGBC and human β cells, we hypothesized that the surface antigens found in human islets may be induced in rGBC as well. The availability of a pan-islet surface antibody (Hpi1) enabled us to further enrich for the most reprogrammed rGBC, enabling us to subdivide rGBC into two subpopulations: (a) highly β-like Hpi1+ rGBC and (b) poorly reprogrammed Hpi1– rGBC. Of the two, only Hpi1+ rGBCs genetically and phenotypically resembled real β cells, therefore the use of this monoclonal antibody in rGBC (and possibly other reprogrammed cell types) will be useful in ensuring purity of rGBC in future experiments and eventually for transplantation purposes.

Although the rGBC we had so far generated was highly β-like, there are several issues—that remain to be addressed for rGBC to achieve a truly β-cell state and transplantation utility—including the suboptimal reprogramming efficiency (between 10–25%), transient engraftment *in vivo*, presence of residual biliary signature, and persistence of transgenes during reprogramming. The reprogramming rate of less than 25% may be attributed to gene and microRNA sets associated with β cell state that remained deficient in rGBCs. Notably, *ZNF* family of genes encoding for Krueppel C2H2-type zinc-finger family of proteins were well represented (35 out of 44 genes) among the transcription regulators that were highly expressed in human β cells but not in rGBCs. On the other hand, examples of genes implicated in the resistance to reprogramming/insulin induction are *CDX2 [33], FOXO1 [33], HES1 [35], NR5A2 [33],* and *PTF1A* [71]. Based on our transcriptome analysis, primary human GBC expressed significant amounts of *CDX2*, *FOXO1*, *HES1*, and *NR5A*. Even after *M6PN* reprogramming the resulting rGBC still expressed high levels of these four factors, which may warrant silencing of these negative regulators in future reprogramming of GBC. Although microRNAs hsa-miR-3168 and hsa-miR-7-2-3p were upregulated in β cells but not in rGBC, none of the two have proven link to β cell program [72]. Similarly, hsa-miR-31-5p was differentially expressed in rGBC and it remains to be tested whether this non-coding RNA targets critical genes involved in the β cell program.

Vector-encoded transgenes as measured by GFP (which is co-expressed with Ad5-M6P) in rGBC showed continued expression. Ideally, transgene expression like that of *NEUROG3* should be short-lived, since it is not expressed in adult β cells but only transiently early in pancreatic development during endocrine specification [73]. The continued expression of *NEUROG3* may have contributed to the immature and polyhormonal phenotype [74], the finite duration of *INS* expression, and overall viability of rGBC [75]. Given the limited lifespan of rGBCs, we were not able to determine how the temporal withdrawal of the exogenous reprogramming factors will affect expression of endogenous factors and overall stability of rGBC reprogramming. Indirect immunofluorescence staining patterns of NEUROD1, NKX2-2, GHRL, and PP in rGBC are indicative of how persistent transgene expression of *NEUROG3* and heterogeneous viral transduction produce partial reprogramming. Moreover, the mostly cytoplasmic localization of NEUROD1, NKX2-2, and NKX6-1 proteins potentially contribute to the incomplete cell fate conversion of rGBCs into β cells. The cross-regulation of *NKX2-2* and *NEUROD1* are predictive of the type of islet hormones (GHRL, PP, GCG) expressed in *NEUROG3*+ cells [74]. We have noticed the variable GFP intensity in transduced rGBC which suggests that transgene expression of M6PN may be different from cell to cell. Therefore, NEUROG3+ cells have variable transgene expression levels which suggest a heterogeneous population of NEUROG3+ rGBCs resulting to a proportion of immature β phenotype in rGBC.

Recent advances in the differentiation of pluripotent stem cells (PSCs) into functionally glucose-responsive insulin-producing β-like cells have pushed the promise of cell therapy for diabetes closer to reality [11, 14, 15]. However, there are several obstacles that need to be overcome such as an effective and durable transplantation methodology that will protect these PSC-derived β-like cells from host immune response as the widely available stem cell lines are allogeneic. The potential for teratoma formation or uncontrolled proliferation is present and life-long immunosuppression is also assured unless patient-derived PSCs are generated. Even with patient-derived PSCs, some of the disadvantages include the long time required, the complicated and variable nature of reprogramming, expansion, and potential clonal variation, intrapatient variation, and epigenetic abnormalities, and heterogeneous differentiation process to β-like cells [76]. The reprogramming of adult cells like the GBC (albeit not yet as highly efficient as PSC-derived β-like cells) still offers advantages over PSC differentiation: (a) autologous transplantation without the time-consuming and expensive reprogramming to pluripotency and differentiation, and (b) absent teratoma risk (based on greater than six months transplantation of GBC in NSG mice).

In summary, we had developed, for the first time, a reliable and robust culture expansion and genetic reprogramming of multiple unrelated patient-derived adult primary GBCs into pancreatic β-like cells *ex vivo*, thus showing that the human gallbladder is a potentially rich source of reprogrammable cells for autologous cell therapy for diabetes.

## Supporting information

S1 Fig Forced expression of reprogramming transcription factors in human gallbladder cells (GBCs) *in vitro*. Ad5 vectors encoding for *PDX1* (A,B), *NEUROG3* (C,D), *MAFA* (E,F), and *PAX6* (H) were used to transduce GBCs in duplicate culture wells. The transgenes for PDX1, NEUROG3, and MAFA have human codon-optimized sequences. Tricistronic Ad5-PNM was also used for comparison (G). Expression levels of pancreatic endocrine-associated genes were measured on day 4 (A,C,E,G), day 7 (B,D,F), and day 3 (H) posttransduction by RT-qPCR. Relative mRNA expression was measured by determining difference between the Cq values of target genes and reference gene *LAMIN* [2^(-ΔCq)].
(TIFF)

S2 Fig Addition of small molecules to rGBCs *in vitro*. Effects on gene expression of insulin and islet-associated genes by (A) retinoic acid (RA), (B) pre-treatment of DMSO prior to adenoviral transduction, (C) Isoxazole-9 (ISX-9), (D) T3, and (E) SB431542. Intact human islets procured from Integrated Islet Distribution Program (IIDP) were used as controls for RT-qPCR and were not exposed to small molecules.
(TIFF)

S3 Fig Immunofluorescence of rGBCs *in vitro*. (A,B) Immunofluorescence staining of human C-peptide and NKX6-1 (scale bar = 50 μm) (C) Enlarged image of C-peptide and NKX6-1 staining showing double staining (yellow arrows) and nuclear NKX6-1 staining (white arrows). (D-E) Immunostaining for NKX2-2, and NEUROD1 in 2-week old rGBC *in vitro* (scale bar = 20 μm). (F) Enlarged image of C-peptide and nuclear NEUROD1 staining (yellow arrows). (G) PP staining in rGBC (scale bar = 20 μm). (H) GFP expression in rGBC 14 days posttransduction (scale bar = 50 μm).
(TIFF)

S4 Fig Gene expression profile of FACS-sorted Hpi2+/- rGBC populations. (A) Relative gene expression levels of β-associated genes NKX2-2, RFX6, NKX6-1, NEUROD1, and INS in Hpi2 subpopulations relative to unsorted rGBCs and human β cells. (B) Relative transcript levels of other pancreatic endocrine genes SST, GCG, GHRL, TMEM27, and PCSK1 in different Hpi2 subpopulations as measured by RT-qPCR after FACS isolation. Relative expression levels were calculated using the formula: [2^(-ΔCq], where ΔCq = Cq(target gene)-Cq(reference gene LAMIN).
(TIFF)

S5 Fig Global microRNA expression profiles in Hpi1+/- rGBC populations. (A) Correlation matrix of global microRNA expression among the different cell types by plotting the square of Pearson coefficient (R2). (B) Heat map and dendogram of the twenty highest differentially expressed microRNAs enriched in primary GBC and downregulated or absent in human β cells across clustered samples. (C-E) Bland-Altman plots comparing the microRNAs in Hpi1+/- and unsorted rGBC populations to β cells. MicroRNAs near or crossing the threshold broken line are marked denoting microRNAs that were differentially expressed between compared samples. *Additional microRNAs that were differentially expressed between β and Hpi1- rGBC include hsa-miR-191-5p,-26a-1-3p,-182-5p,-20a-3p,-486-3p,-200c-3p.
(TIFF)

S6 Fig Immunofluorescence of rGBC xenografts in NSG mouse model. (A,B) Reprogrammed GBC graft stained for C-peptide, SST (epididymal fat pad), and NEUROD1 (kidney) (Scale bar = 20 μm). (C) Mouse CD31+ cells (red) are found within the area of the rGBC xenograft (marked green) (Scale bar = 200 μm). (D) Reprogrammed GBC (green) co-cultured for 5 days with HUVEC and MSC formed tissue-like structure in vitro (Scale bar = 2 mm). (E) RT-qPCR analysis of genes expressed in rGBC in the presence or absence of HUVEC and MSC. (F) Glucose-stimulated insulin secretion in rGBC in the presence or absence of HUVEC and MSC by measurement of C-peptide released in the supernatants after 2 hours in 1 mM and 25 mM glucose. Fold-change ratios were calculated by using the values obtained from 1 mM glucose exposure as denominator for each group. (G,H,I) Two-week old grafts of rGBC-HUVEC-MSC in NSG kidney (n = 11) and stained for human C-peptide, CD31 (HUVEC marker), and CD44 (MSC marker) (Scale bar = 50 μm).
(TIFF)

S1 Table RT-qPCR primers. (DOCX)

S2 Table Antibodies used for immunofluorescence or flow cytometry. (DOCX)

S3 Table Gene set investigation of the top 224 differentially expressed genes in human beta cells (log_2_FC>5, *p*<0.01) compared to GBC that overlaps with canonical pathways, BioCarta, KEGG, REACTOME, and gene ontology gene sets using molecular signature database. (DOCX)

S4 Table Gene set investigation of the top 151 differentially expressed genes shared by human beta cells and rGBCs (log_2_FC>5, *p*<0.01) compared to GBC that overlaps with canonical pathways, BioCarta, KEGG, REACTOME, and gene ontology gene sets using molecular signature database. (DOCX)

S5 Table Gene set investigation of 993 “Beta genes” that remain uninduced in rGBC (log_2_FC15, *p*<0.01) when compared to human beta cells to determine the molecular signatures of gene sets characteristic of rGBC potentially needed to be upregulated for a more efficient genetic reprogramming. (DOCX)

S6 Table Gene set investigation of 1809 “GB genes” upregulated in rGBC (*i*.*e*. absent or downregulated in human beta cells). (DOCX)

S7 Table Gene set investigation of the 248 “Beta genes” upregulated in Hpi1+ rGBC and not found in Hpi1- and unsorted rGBC. (DOCX)

S8 Table Gene set investigation of the top 119 “GB genes” upregulated in Hpi1+ rGBC (log_2_FC>5, *p*<0.01) to determine the molecular signatures of gene sets characteristic of unreprogrammed GBC potentially needed to be downregulated for a more efficient genetic reprogramming to beta cells. (DOCX)

S9 Table ARRIVE guideline checklist. (PDF)
