# Supplementary figures and images for: Reprogramming human gallbladder cells into insulin-producing β-like cells
Source: PLoS One. 2017 Aug 16;12(8):e0181812. doi: 10.1371/journal.pone.0181812 (PMC5558938; doi:10.1371/journal.pone.0181812)

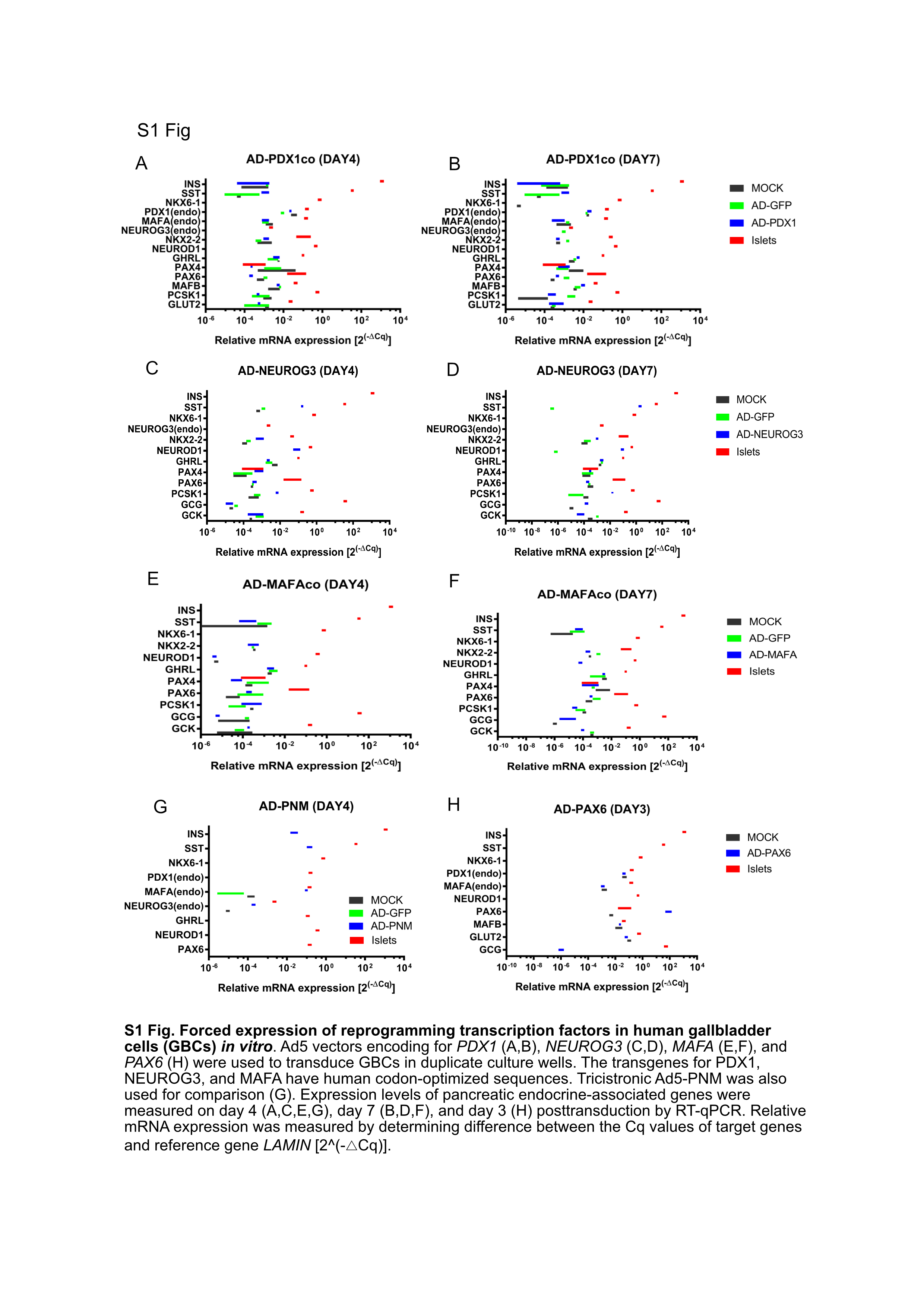

Supplement: S1 Fig — Ad5 vectors encoding for PDX1 (A,B), NEUROG3 (C,D), MAFA (E,F), and PAX6 (H) were used to transduce GBCs in duplicate culture wells. The transgenes for PDX1, NEUROG3, and MAFA have human codon-optimized sequences. Tricistronic Ad5-PNM was also used for comparison (G). Expression levels of pancreatic endocrine-associated genes were measured on day 4 (A,C,E,G), day 7 (B,D,F), and day 3 (H) posttransduction by RT-qPCR. Relative mRNA expression was measured by determining difference between the Cq values of target genes and reference gene LAMIN [2^(-ΔCq)]. (TIFF) [file pone.0181812.s001.tiff]

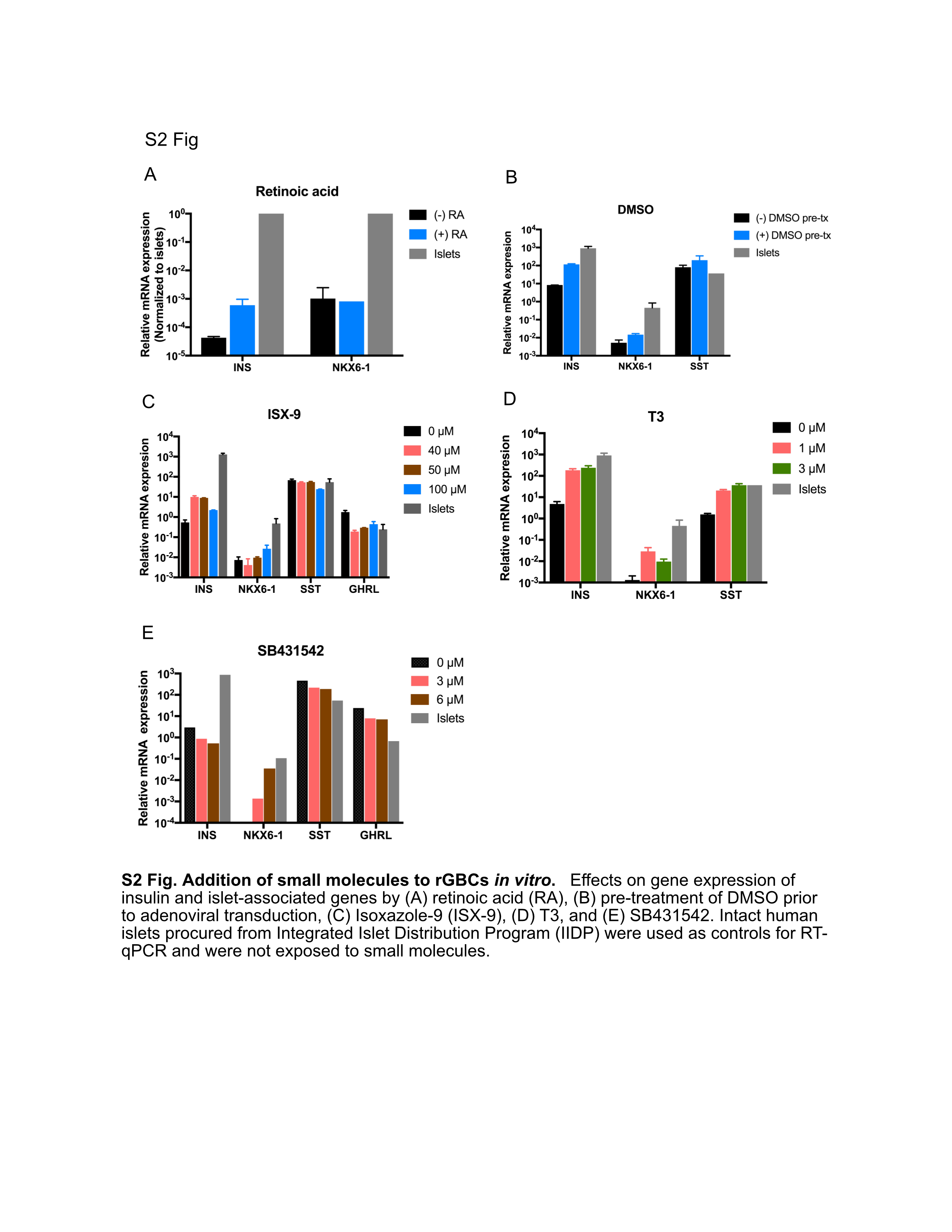

Supplement: S2 Fig — Effects on gene expression of insulin and islet-associated genes by (A) retinoic acid (RA), (B) pre-treatment of DMSO prior to adenoviral transduction, (C) Isoxazole-9 (ISX-9), (D) T3, and (E) SB431542. Intact human islets procured from Integrated Islet Distribution Program (IIDP) were used as controls for RT-qPCR and were not exposed to small molecules. (TIFF) [file pone.0181812.s002.tiff]

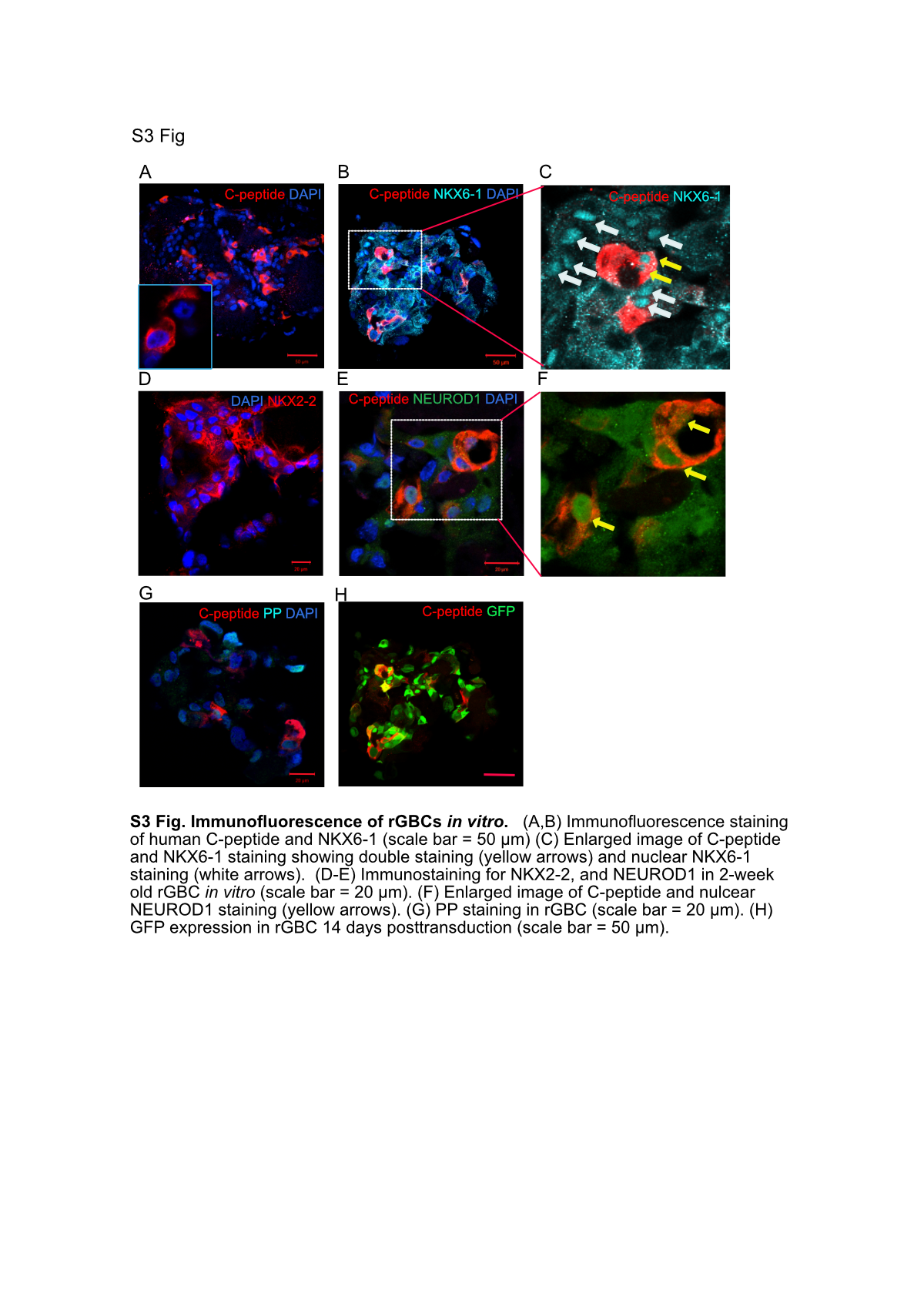

Supplement: S3 Fig — (A,B) Immunofluorescence staining of human C-peptide and NKX6-1 (scale bar = 50 μm) (C) Enlarged image of C-peptide and NKX6-1 staining showing double staining (yellow arrows) and nuclear NKX6-1 staining (white arrows). (D-E) Immunostaining for NKX2-2, and NEUROD1 in 2-week old rGBC in vitro (scale bar = 20 μm). (F) Enlarged image of C-peptide and nuclear NEUROD1 staining (yellow arrows). (G) PP staining in rGBC (scale bar = 20 μm). (H) GFP expression in rGBC 14 days posttransduction (scale bar = 50 μm). (TIFF) [file pone.0181812.s003.tiff]

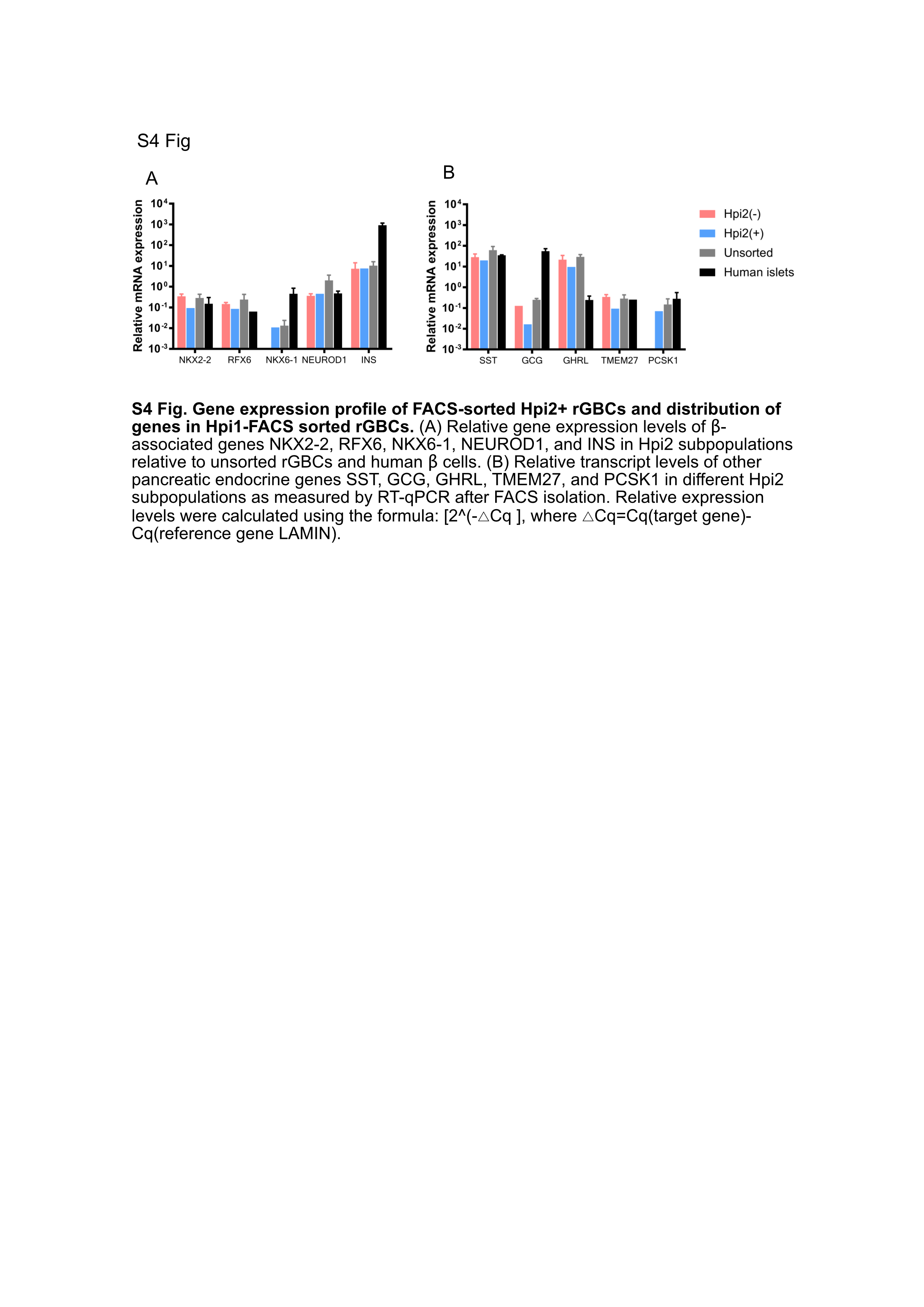

Supplement: S4 Fig — (A) Relative gene expression levels of β-associated genes NKX2-2, RFX6, NKX6-1, NEUROD1, and INS in Hpi2 subpopulations relative to unsorted rGBCs and human β cells. (B) Relative transcript levels of other pancreatic endocrine genes SST, GCG, GHRL, TMEM27, and PCSK1 in different Hpi2 subpopulations as measured by RT-qPCR after FACS isolation. Relative expression levels were calculated using the formula: [2^(-ΔCq], where ΔCq = Cq(target gene)-Cq(reference gene LAMIN). (TIFF) [file pone.0181812.s004.tiff]

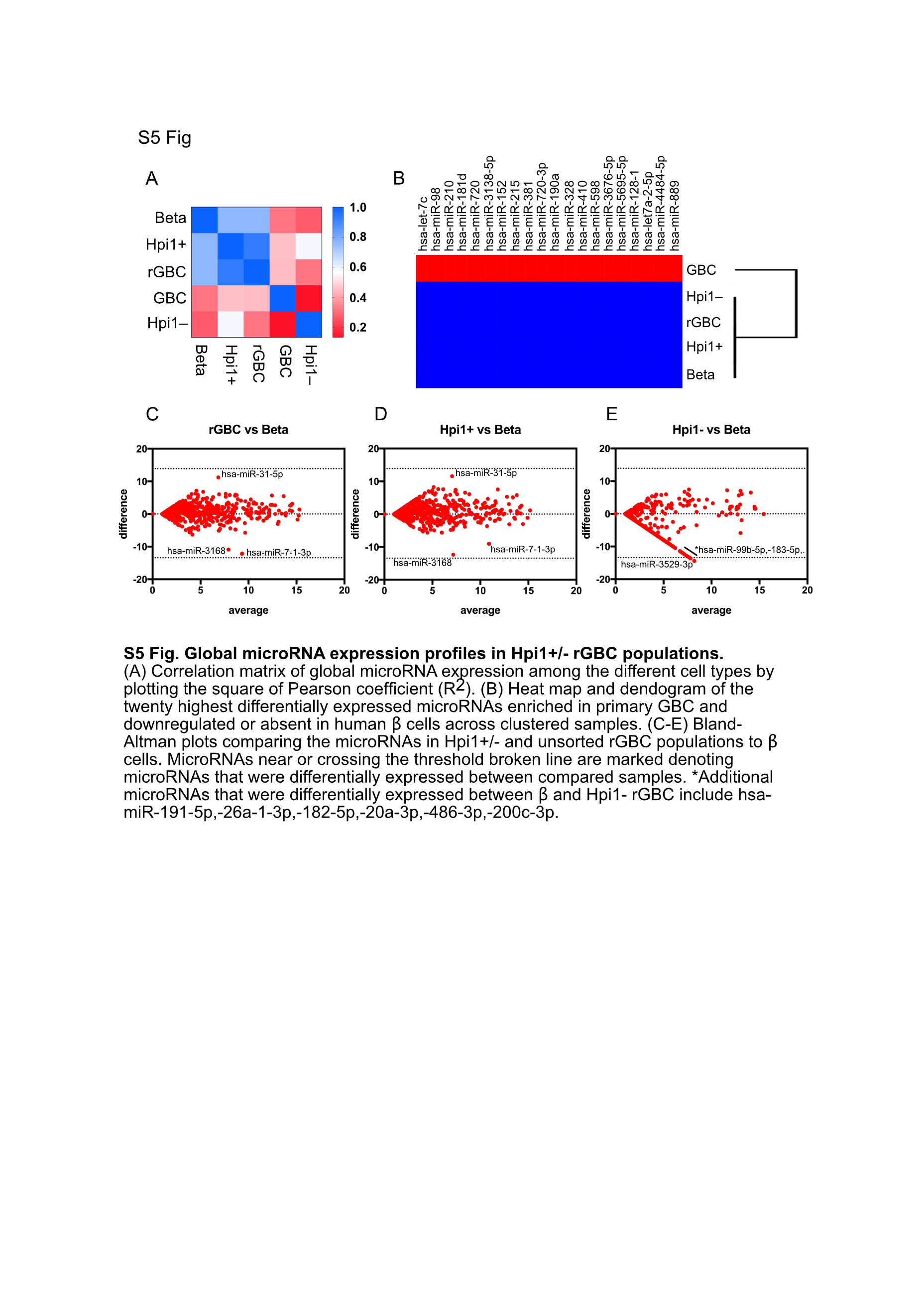

Supplement: S5 Fig — (A) Correlation matrix of global microRNA expression among the different cell types by plotting the square of Pearson coefficient (R2). (B) Heat map and dendogram of the twenty highest differentially expressed microRNAs enriched in primary GBC and downregulated or absent in human β cells across clustered samples. (C-E) Bland-Altman plots comparing the microRNAs in Hpi1+/- and unsorted rGBC populations to β cells. MicroRNAs near or crossing the threshold broken line are marked denoting microRNAs that were differentially expressed between compared samples. *Additional microRNAs that were differentially expressed between β and Hpi1- rGBC include hsa-miR-191-5p,-26a-1-3p,-182-5p,-20a-3p,-486-3p,-200c-3p. (TIFF) [file pone.0181812.s005.tiff]

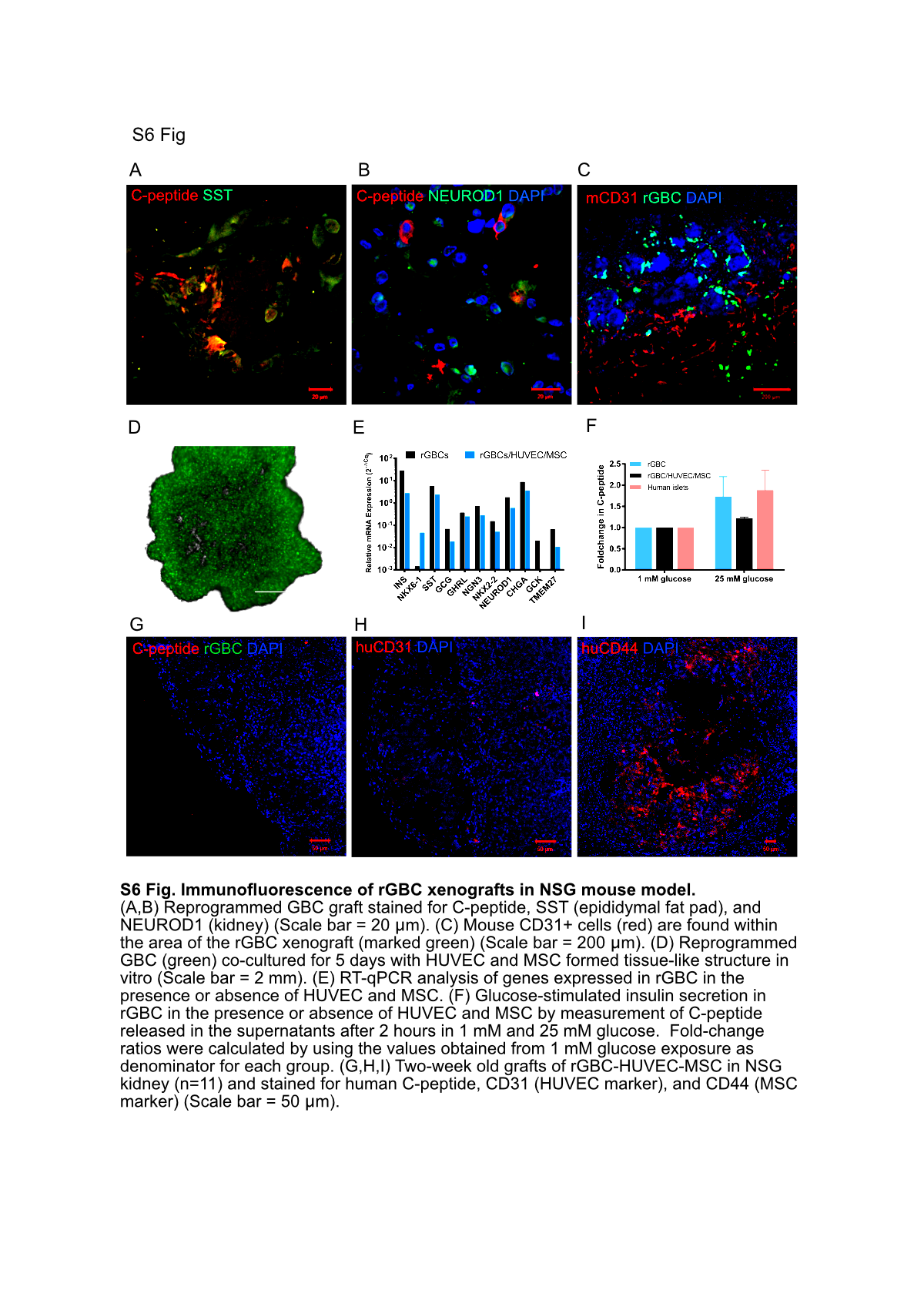

Supplement: S6 Fig — (A,B) Reprogrammed GBC graft stained for C-peptide, SST (epididymal fat pad), and NEUROD1 (kidney) (Scale bar = 20 μm). (C) Mouse CD31+ cells (red) are found within the area of the rGBC xenograft (marked green) (Scale bar = 200 μm). (D) Reprogrammed GBC (green) co-cultured for 5 days with HUVEC and MSC formed tissue-like structure in vitro (Scale bar = 2 mm). (E) RT-qPCR analysis of genes expressed in rGBC in the presence or absence of HUVEC and MSC. (F) Glucose-stimulated insulin secretion in rGBC in the presence or absence of HUVEC and MSC by measurement of C-peptide released in the supernatants after 2 hours in 1 mM and 25 mM glucose. Fold-change ratios were calculated by using the values obtained from 1 mM glucose exposure as denominator for each group. (G,H,I) Two-week old grafts of rGBC-HUVEC-MSC in NSG kidney (n = 11) and stained for human C-peptide, CD31 (HUVEC marker), and CD44 (MSC marker) (Scale bar = 50 μm). (TIFF) [file pone.0181812.s006.tiff]
