# Supplementary material for: Reprogramming human gallbladder cells into insulin-producing β-like cells
Source: PLoS One. 2017 Aug 16;12(8):e0181812. doi: 10.1371/journal.pone.0181812 (PMC5558938; doi:10.1371/journal.pone.0181812)
Supplement: S1 Table — (DOCX) [file pone.0181812.s007.docx]

**S1 Table. RT-qPCR primers**

| **GENE** | **Forward Primer (5’-3’)** | **Reverse Primer (5’-3’)** |
| --- | --- | --- |
| *ABCC8* | cctggtccccagtgcagatg | ccacctgccccacgatcata |
| *CHGA* | gcaaaccgcagaccagagga | ccgtagtgcctgcagctggt |
| *GCK* | cgcggtccatctccttctgc | aggctgaggggtcccagctc |
| *GHRL* | ttcaacgccccctttgatgttg | gcctctttggcctcttcccaga |
| *GCG* | agcttcccaggcagacccactc | atggcgcttgtcctcgttcatc |
| *GLUT2* | ggcagggcgacgttctctcttt | cccactgacatgaagatggcaca |
| *HOPX* | acccccgcgcttccttcactccttcct | ggtcctctgtggggccgctcgcg |
| *INSULIN* | cgcagcctttgtgaaccaacac | aagcctcgttccccgcacacta |
| *KCNJ11* | catgatcatcagcgccacca | gccgttctccatggggatgt |
| *LAMINA* | agatgcgggcaaggatgcag | cctcctcgccctccaagagc |
| *MAFA* | accacgtgcgcctggaggagcgct | tgcgccgcttctgcttgagccgga |
| *MAFB* | gagaaactcgccaactccggct | accggccacgactcacagaaag |
| *MUC5B* | ctgggagaatgcagggcaca | ggctcaggctggggaagaca |
| *NEUROD1* | ggaggccccagggttatgaga | tctgcctcgtgctcctcgtc |
| *NEUROG3* | actgagcaagcagcgggagt | cgtccagtgccgagttgaggtt |
| *NKX2-2* | gcagagcctgcccctgaaga | gctccggggacttggagctt |
| *NKX6-1* | gggctcgtttggcctattcg | gcagcgtgcttcttcctcca |
| *PAX4* | cacaggcgggcagggcagtggga | ctgccgggtatccagaggcaggggc |
| *PAX6* | gcgctctgccgcctatgcccagct | aggggaaatgagtcctgttgaagtggtgcccg |
| *PCSK1* | ccggagggatgagttggaggag | tggtgattgctttggcggtgag |
| *PDX1* | tggaggagcccaaccgcgtccagc | gcgccgcctgcccactggcctt |
| *PPY* | tctccctgctgctcctgtccac | ccatctgctctggtgtggcatt |
| *RFX6* | gccagctgccaaggagcaac | gggggcagtgaacgaatgct |
| *SST* | gctgctgtctgaacccaaccaga | tcaagcctcatttcatcctgctca |
| *SYP* | gcggacatggacgtggtga | tgtggcaaaggcgaagatgg |
| *TMEM27* | ctggtgactgccattcatgctg | ccaggcatatgctttatctcccaga |
