# Supplementary material for: Reprogramming human gallbladder cells into insulin-producing β-like cells
Source: PLoS One. 2017 Aug 16;12(8):e0181812. doi: 10.1371/journal.pone.0181812 (PMC5558938; doi:10.1371/journal.pone.0181812)
Supplement: S2 Table — (DOCX) [file pone.0181812.s008.docx]

**S2 Table. Antibodies used for immunofluorescence or flow cytometry**

| **Antigen** | **Host/Class/type (clone)** | **Source** | **Product code** |
| --- | --- | --- | --- |
| CD31 | Mouse mAb IgG2a (M89D3) | BD Biosciences | 558068 |
| CD44 | Mouse mAb IgG2b (G44-26) | BD Biosciences | 560533 |
| C-peptide | Rat mAb IgG2a  (GN-ID4) | DSHB, Iowa | GN-ID4 |
| EpCAM | Mouse mAb IgG1  (Ber-EP4) | Dako | F0860 |
| Ghrelin | Rabbit pAb IgG  (H-40) | Sta. Cruz Biotechnology | sc-50297 |
| Glucagon | Rabbit pAb | Dako | A0565 |
| Hpi1 (human pancreatic islet) | Mouse mAb IgG1 (HIC0-4F9) | Novus Biologicals | NBP1-18872 |
| Hpi2 (human pancreatic islet) | Mouse mAb IgG1 (HIC1-2B4) | Novus Biologicals | NBP1-18946 |
| Mitochondria | Mouse mAb IgG1  (113-1) | Abcam | ab92824 |
| Mouse CD31 | Rat mAb IgG2a (MEC13.3) | BD Biosciences | 550274 |
| NeuroD | Goat pAb IgG | Sta. Cruz Biotechnology | sc-1084 |
| Nkx-2.2 | Rabbit pAb IgG  (H-60) | Sta. Cruz Biotechnology | sc-25404 |
| NKX6-1 | Rabbit pAb | Sigma | HPA036774 |
| Pancreatic polypeptide (PP) | Rabbit pAb | Abcam | ab16003 |
| Somatostatin | Goat pAb IgG  (D-20) | Sta. Cruz Biotechnology | sc-7819 |
