# Supplementary material for: Reprogramming human gallbladder cells into insulin-producing β-like cells
Source: PLoS One. 2017 Aug 16;12(8):e0181812. doi: 10.1371/journal.pone.0181812 (PMC5558938; doi:10.1371/journal.pone.0181812)
Supplement: S3 Table — (DOCX) [file pone.0181812.s009.docx]

**S3 Table. Gene set investigation of the top 224 differentially expressed genes in human beta cells (log_2_FC>5, *p*<0.01) compared to GBC that overlaps with Canonical pathways, BioCarta, KEGG, REACTOME, and Gene Ontology gene sets using Molecular Signature Database**

| **Gene Set Name** | **#Genes in Gene Set (K)** | **Description** | **#Genes in Overlap (k)** | **k/K** | **p-value** | **FDR q-value** |
| --- | --- | --- | --- | --- | --- | --- |
| REACTOME_REGULATION OF_INSULIN_SECRETION | 93 | Genes involved in Regulation of Insulin Secretion | 12 | 0.129 | 3.93E-14 | 1.10E-10 |
| REACTOME_INTEGRATION OF_ENERGY_METABOLISM | 120 | Genes involved in Integration of energy metabolism | 12 | 0.1 | 8.87E-13 | 1.14E-09 |
| REACTOME_REGULATION OF_BETA_CELL_DEVELOPMENT | 30 | Genes involved in Regulation of beta-cell development | 8 | 0.2667 | 1.50E-12 | 1.14E-09 |
| EXTRACELLULAR_SPACE | 245 | Genes annotated by the GO term GO:0005615 | 15 | 0.0612 | 1.64E-12 | 1.14E-09 |
| REACTOME_REGULATION OF_GENE_EXPRESSION_IN_BETA_CELLS | 20 | Genes involved in Regulation of gene expression in beta cells | 7 | 0.35 | 4.37E-12 | 2.43E-09 |
| PEPTIDASE_ACTIVITY | 176 | Genes annotated by the GO term GO:0008233 | 12 | 0.0682 | 8.33E-11 | 3.87E-08 |
| EXTRACELLULAR_REGION_PART | 338 | Genes annotated by the GO term GO:0044421 | 15 | 0.0444 | 1.56E-10 | 6.19E-08 |
| CELL_CELL_SIGNALING | 404 | Genes annotated by the GO term GO:0007267 | 16 | 0.0396 | 2.00E-10 | 6.94E-08 |
| EXTRACELLULAR_REGION | 447 | Genes annotated by the GO term GO:0005576 | 16 | 0.0358 | 8.62E-10 | 2.67E-07 |
| KEGG_MATURITY_ONSET_DIABETES_OF_THE_YOUNG | 25 | Maturity onset diabetes of the young | 6 | 0.24 | 2.06E-09 | 5.72E-07 |
| ESTABLISHMENT_OF_LOCALIZATION | 870 | Genes annotated by the GO term GO:0051234 | 21 | 0.0241 | 2.39E-09 | 6.04E-07 |
| REGULATION_OF_BIOLOGICAL_QUALITY | 419 | Genes annotated by the GO term GO:0065008 | 15 | 0.0358 | 2.94E-09 | 6.81E-07 |
| NABA_MATRISOME_ASSOCIATED | 753 | Ensemble of genes encoding ECM-associated proteins including ECM-affiliated proteins, ECM regulators and secreted factors | 19 | 0.0252 | 7.02E-09 | 1.50E-06 |
| REACTOME_NEURONAL_SYSTEM | 279 | Genes involved in Neuronal System | 12 | 0.043 | 1.56E-08 | 3.10E-06 |
| CELL_SURFACE_RECEPTOR_LINKED SIGNAL_TRANSDUCTION_GO_0007166 | 641 | Genes annotated by the GO term GO:0007166 | 17 | 0.0265 | 2.19E-08 | 3.88E-06 |
| SYSTEM_PROCESS | 563 | Genes annotated by the GO term GO:0003008 | 16 | 0.0284 | 2.23E-08 | 3.88E-06 |
| NABA_MATRISOME | 1028 | Ensemble of genes encoding extracellular matrix and extracellular matrix-associated proteins | 21 | 0.0204 | 4.20E-08 | 6.88E-06 |
| REACTOME_INSULIN_SYNTHESIS_AND_PROCESSING | 21 | Genes involved in Insulin Synthesis and Processing | 5 | 0.2381 | 5.02E-08 | 7.77E-06 |
| DIGESTION | 44 | Genes annotated by the GO term GO:0007586 | 6 | 0.1364 | 7.58E-08 | 1.06E-05 |
| HORMONE_ACTIVITY | 44 | Genes annotated by the GO term GO:0005179 | 6 | 0.1364 | 7.58E-08 | 1.06E-05 |
