# Supplementary material for: Reprogramming human gallbladder cells into insulin-producing β-like cells
Source: PLoS One. 2017 Aug 16;12(8):e0181812. doi: 10.1371/journal.pone.0181812 (PMC5558938; doi:10.1371/journal.pone.0181812)
Supplement: S4 Table — (DOCX) [file pone.0181812.s010.docx]

**S4 Table. Gene set investigation of the top 151 differentially expressed genes shared by human beta cells and rGBCs (log_2_FC>5, *p*<0.01) compared to GBC that overlaps with Canonical pathways, BioCarta, KEGG, REACTOME, and Gene Ontology gene sets using Molecular Signature Database**

| **Gene Set Name** | **#Genes in Gene Set (K)** | **Description** | **#Genes in Overlap (k)** | **k/K** | **p-value** | **FDR q-value** |
| --- | --- | --- | --- | --- | --- | --- |
| REACTOME_REGULATION OF_ BETA_CELL_DEVELOPMENT | 30 | Genes involved in Regulation of beta-cell development | 7 | 0.2333 | 6.87E-12 | 1.91E-08 |
| REACTOME_REGULATION OF_GENE_ EXPRESSION_IN_BETA_CELLS | 20 | Genes involved in Regulation of gene expression in beta cells | 6 | 0.3 | 4.25E-11 | 5.91E-08 |
| KEGG_TYPE_I_DIABETES_MELLITUS | 44 | Type I Diabetes Mellitus | 7 | 0.1591 | 1.24E-10 | 1.15E-07 |
| MEMBRANE | 1994 | Genes annotated by the GO term GO:0016020 | 27 | 0.0135 | 3.90E-10 | 2.72E-07 |
| REACTOME_DEVELOPMENTAL_BIOLOGY | 396 | Genes involved in Developmental Biology | 13 | 0.0328 | 8.14E-10 | 4.53E-07 |
| MULTICELLULAR_ORGANISMAL_ DEVELOPMENT | 1049 | Genes annotated by the GO term GO:0007275 | 19 | 0.0181 | 2.05E-09 | 9.51E-07 |
| EXTRACELLULAR_REGION | 447 | Genes annotated by the GO term GO:0005576 | 13 | 0.0291 | 3.49E-09 | 1.39E-06 |
| SIGNAL_TRANSDUCTION | 1634 | Genes annotated by the GO term GO:0007165 | 23 | 0.0141 | 4.45E-09 | 1.55E-06 |
| KEGG_MATURITY_ONSET_DIABETES_ OF_THE_YOUNG | 25 | Maturity onset diabetes of the young | 5 | 0.2 | 1.81E-08 | 5.58E-06 |
| SYSTEM_DEVELOPMENT | 861 | Genes annotated by the GO term GO:0048731 | 16 | 0.0186 | 2.93E-08 | 7.85E-06 |
| MEMBRANE_PART | 1670 | Genes annotated by the GO term GO:0044425 | 22 | 0.0132 | 3.23E-08 | 7.85E-06 |
| ESTABLISHMENT_OF_LOCALIZATION | 870 | Genes annotated by the GO term GO:0051234 | 16 | 0.0184 | 3.38E-08 | 7.85E-06 |
| TRANSPORT | 795 | Genes annotated by the GO term GO:0006810 | 15 | 0.0189 | 6.68E-08 | 1.43E-05 |
| INTRINSIC_TO_MEMBRANE | 1348 | Genes annotated by the GO term GO:0031224 | 19 | 0.0141 | 1.09E-07 | 2.16E-05 |
| PLASMA_MEMBRANE | 1426 | Genes annotated by the GO term GO:0005886 | 19 | 0.0133 | 2.57E-07 | 4.60E-05 |
| ANATOMICAL_STRUCTURE_DEVELOPMENT | 1013 | Genes annotated by the GO term GO:0048856 | 16 | 0.0158 | 2.64E-07 | 4.60E-05 |
| ESTABLISHMENT_OF_CELLULAR_LOCALIZATION | 353 | Genes annotated by the GO term GO:0051649 | 10 | 0.0283 | 3.03E-07 | 4.96E-05 |
| NABA_MATRISOME | 1028 | Ensemble of genes encoding extracellular matrix and extracellular matrix-associated proteins | 16 | 0.0156 | 3.21E-07 | 4.97E-05 |
| INTEGRAL_TO_MEMBRANE | 1330 | Genes annotated by the GO term GO:0016021 | 18 | 0.0135 | 4.35E-07 | 6.37E-05 |
| CELLULAR_LOCALIZATION | 371 | Genes annotated by the GO term GO:0051641 | 10 | 0.027 | 4.76E-07 | 6.63E-05 |
