# Supplementary material for: Reprogramming human gallbladder cells into insulin-producing β-like cells
Source: PLoS One. 2017 Aug 16;12(8):e0181812. doi: 10.1371/journal.pone.0181812 (PMC5558938; doi:10.1371/journal.pone.0181812)
Supplement: S5 Table — (DOCX) [file pone.0181812.s011.docx]

**S5 Table. Gene set investigation of 993 “Beta genes” that remain uninduced in rGBC (log_2_FC15, *p*<0.01) when compared to human beta cells to determine the molecular signatures of gene sets characteristic of rGBC potentially needed to be upregulated for a more efficient genetic reprogramming.**

| **Gene Set Name** | **#Genes in Gene Set (K)** | **Description** | **#Genes in Overlap (k)** | **k/K** | **p-value** | **FDR q-value** |
| --- | --- | --- | --- | --- | --- | --- |
| REACTOME_GENERIC_TRANSCRIPTION_PATHWAY | 352 | Genes involved in Generic Transcription Pathway | 44 | 0.0125 | 4.74E-21 | 1.32E-17 |
| NABA_MATRISOME | 1028 | Ensemble of genes encoding extracellular matrix-associated proteins | 72 | 0.07 | 1.39E-18 | 1.93E-15 |
| MEMBRANE | 1994 | Genes annotated by the GO term GO:0016020 | 102 | 0.0512 | 3.20E-16 | 2.97E-13 |
| INTRINSIC_TO_MEMBRANE | 1348 | Genes annotated by the GO term GO:0031224 | 79 | 0.0586 | 7.09E-16 | 4.94E-13 |
| INTEGRAL_TO_MEMBRANE | 1330 | Genes annotated by the GO term GO:0016021 | 77 | 0.0579 | 3.21E-15 | 1.79E-12 |
| ANATOMICAL_STRUCTURE_ DEVELOPMENT | 1013 | Genes annotated by the GO term GO:0048856 | 65 | 0.0642 | 4.58E-15 | 2.12E-12 |
| MEMBRANE_PART | 1670 | Genes annotated by the GO term GO:0044425 | 88 | 0.0527 | 7.59E-15 | 3.02E-12 |
| PLASMA_MEMBRANE | 1426 | Genes annotated by the GO term GO:0005886 | 79 | 0.0554 | 1.45E-14 | 5.06E-12 |
| SYSTEM_DEVELOPMENT | 861 | Genes annotated by the GO term GO:0048731 | 58 | 0.0674 | 1.82E-14 | 5.61E-12 |
| MULTICELLULAR_ORGANISMAL_DEVELOPMENT | 1049 | Genes annotated by the GO term GO:0007275 | 65 | 0.062 | 2.32E-14 | 6.46E-12 |
| SIGNAL_TRANSDUCTION | 1634 | Genes annotated by the GO term GO:0007165 | 85 | 0.052 | 4.58E-14 | 1.16E-11 |
| EXTRACELLULAR_REGION | 447 | Genes annotated by the GO term GO:0005576 | 39 | 0.0872 | 1.45E-13 | 3.36E-11 |
| PLASMA_MEMBRANE_PART | 1158 | Genes annotated by the GO term GO:0044459 | 67 | 0.0579 | 2.23E-13 | 4.78E-11 |
| INTRINSIC_TO_PLASMA_MEMBRANE | 991 | Genes annotated by the GO term GO:0031226 | 59 | 0.0595 | 1.98E-12 | 3.93E-10 |
| INTEGRAL_TO_PLASMA_MEMBRANE | 977 | Genes annotated by the GO term GO:0005887 | 58 | 0.0594 | 3.43E-12 | 6.36E-10 |
| REGULATION_OF_BIOLOGICAL_QUALITY | 419 | Genes annotated by the GO term GO:0065008 | 33 | 0.0788 | 1.61E-10 | 2.81E-08 |
| NABA_MATRISOME_ASSOCIATED | 753 | Ensemble of genes encoding ECM-associated proteins including ECM-affiliated proteins, ECM regulators and secreted factors | 46 | 0.0611 | 2.44E-10 | 3.99E-08 |
| EXTRACELULAR_REGION_PART | 338 | Genes annotated by the GO term GO:0044421 | 29 | 0.0858 | 2.84E-10 | 4.29E-08 |
| NABA_CORE_MATRISOME | 275 | Ensemble of genes encoding core extracellular matrix including ECM glycoproteins, collagens and proteoglycans | 26 | 0.0945 | 2.93E-10 | 4.29E-08 |
| NERVOUS_SYSTEM_DEVELOPMENT | 385 | Genes annotated by the GO term GO:0007399 | 31 | 0.0805 | 3.36E-10 | 4.67E-08 |
