# Supplementary material for: Reprogramming human gallbladder cells into insulin-producing β-like cells
Source: PLoS One. 2017 Aug 16;12(8):e0181812. doi: 10.1371/journal.pone.0181812 (PMC5558938; doi:10.1371/journal.pone.0181812)
Supplement: S6 Table — (DOCX) [file pone.0181812.s012.docx]

**S6 Table. Gene set investigation of 1809 “GB genes” upregulated in rGBC (*i.e.* absent or downregulated in human beta cells).**

| **Gene Set Name** | **#Genes in Gene Set (K)** | **Description** | **#Genes Overlap (k)** | **k/K** | **p-value** | **FDR q-value** |
| --- | --- | --- | --- | --- | --- | --- |
| GO_SMALL_MOLECULE_METABOLIC_PROCESS | 1767 | The chemical reactions and pathways involving small molecules | 257 | 0.1454 | 8.31E-77 | 6.23E-73 |
| GO_ENZYME_BINDING | 1737 | Interacting selectively and non-covalently with any enzyme | 252 | 0.1451 | 5.08E-75 | 1.90E-71 |
| GO_CELL_JUNCTION | 1151 | A cellular component that forms a specialized region of connection between two or more cells/extracellular matrix | 197 | 0.1712 | 2.25E-70 | 5.61E-67 |
| GO_ORGANONITROGEN_COMPOUND_ METABOLIC_PROCESS | 1796 | The organonitrogen chemical reactions and pathways | 246 | 0.137 | 4.32E-68 | 8.09E-65 |
| GO_MEMBRANE_REGION | 1134 | A membrane that is a part of a larger membrane | 192 | 0.1693 | 9.26E-68 | 1.39E-64 |
| GO_TISSUE_DEVELOPMENT | 1518 | The process whose specific outcome is the progression of a tissue over time, from its formation to the mature structure | 218 | 0.1436 | 8.67E-64 | 1.08E-60 |
| GO_IMMUNE_SYSTEM_PROCESS | 1984 | Any process involved in the development or functioning of the immune system | 250 | 0.126 | 5.57E-62 | 5.96E-59 |
| GO_CYTOSKELETON | 1967 | Any of the various filamentous elements that form the internal framework of cells | 245 | 0.1246 | 9.99E-60 | 9.36E-57 |
| GO_POSITIVE_REGULATION_OF_ MOLECULAR_FUNCTION | 1791 | Any process that activates or increases the rate or extent of a molecular function | 227 | 0.1267 | 1.52E-56 | 1.27E-53 |
| GO_GOLGI_APPARATUS | 1445 | A compound membranous cytoplasmic organelle of eukaryotic cells | 201 | 0.1391 | 2.04E-56 | 1.53E-53 |
| GO_POSITIVE_REGULATION_OF_ RESPONSE_TO_STIMULUS | 1929 | Any process that activates, maintains or increases the rate of a response to a stimulus | 235 | 0.1218 | 1.89E-55 | 1.29E-52 |
| GO_CELLULAR_RESPONSE_TO_ORGANIC_ SUBSTANCE | 1848 | Any process that results in a change in state or activity of a cell | 229 | 0.1239 | 2.65E-55 | 1.66E-52 |
| GO_PHOSPHATE_CONTAINING_ COMPOUND_METABOLIC_PROCESS | 1977 | The chemical reactions and pathways involving the phosphate group, the anion or salt of any phosphoric acid. | 238 | 0.1204 | 3.10E-55 | 1.79E-52 |
| GO_ANCHORING_JUNCTION | 489 | A cell junction that mechanically attaches a cell (and its cytoskeleton) to neighboring cells or to the extracellular matrix. | 114 | 0.2331 | 6.33E-55 | 3.39E-52 |
| GO_REGULATION_OF_TRANSPORT | 1804 | Any process that modulates the frequency, rate or extent of the directed movement of substances | 224 | 0.1242 | 3.26E-54 | 1.63E-51 |
| GO_REGULATION_OF_CELL_DEATH | 1472 | Any process that modulates the rate or frequency of cell death | 199 | 0.1352 | 7.57E-54 | 3.55E-51 |
| GO_PLASMA_MEMBRANE_REGION | 929 | A membrane that is a (regional) part of the plasma membrane | 154 | 0.1658 | 5.24E-53 | 2.31E-50 |
| GO_RESPONSE_TO_EXTERNAL_STIMULUS | 1821 | Any process that results in a change in state or activity of a cell or an organism as a result of an external stimulus | 222 | 0.1219 | 2.39E-52 | 9.97E-50 |
| GO_EPITHELIUM_DEVELOPMENT | 945 | Formation and maturation of an epithelium | 154 | 0.163 | 5.14E-52 | 2.03E-49 |
| GO_REGULATION_OF_INTRACELLULAR_ SIGNAL_TRANSDUCTION | 1656 | Any process that modulates the frequency, rate or extent of intracellular signal transduction | 209 | 0.1262 | 1.19E-51 | 4.47E-49 |
