# Supplementary material for: Reprogramming human gallbladder cells into insulin-producing β-like cells
Source: PLoS One. 2017 Aug 16;12(8):e0181812. doi: 10.1371/journal.pone.0181812 (PMC5558938; doi:10.1371/journal.pone.0181812)
Supplement: S7 Table — (DOCX) [file pone.0181812.s013.docx]

**S7 Table. Gene set investigation of the 248 “Beta genes” upregulated in Hpi1+ rGBC and not found in Hpi1- and unsorted rGBC.**

| **Gene Set Name** | **#Genes in Gene Set (K)** | **Description** | **#Genes in Overlap (k)** | **k/K** | **p-value** | **FDR q-value** |
| --- | --- | --- | --- | --- | --- | --- |
| NERVOUS_SYSTEM_DEVELOPMENT | 385 | Genes annotated by the GO term GO:0007399 | 14 | 0.0364 | 2.55E-08 | 4.60E-05 |
| ANATOMICAL_STRUCTURE_DEVELOPMENT | 1013 | Genes annotated by the GO term GO:0048856 | 22 | 0.0217 | 3.38E-08 | 4.60E-05 |
| SYSTEM_DEVELOPMENT | 861 | Genes annotated by the GO term GO:0048731 | 20 | 0.0232 | 4.96E-08 | 4.60E-05 |
| MULTICELLULAR_ORGANISMAL_DEVELOPMENT | 1049 | Genes annotated by the GO term GO:0007275 | 21 | 0.02 | 2.69E-07 | 1.87E-04 |
| REACTOME_NEURONAL_SYSTEM | 279 | Genes involved in Neuronal System | 10 | 0.0358 | 2.95E-06 | 1.20E-03 |
| AXONOGENESIS | 43 | Genes annotated by the GO term GO:0007409 | 5 | 0.1163 | 3.37E-06 | 1.20E-03 |
| REACTOME_VOLTAGE_GATED_POTASSIUM_ CHANNELS | 43 | Genes involved in Voltage-gated Potassium channels | 5 | 0.1163 | 3.37E-06 | 1.20E-03 |
| NEURON_DIFFERENTIATION | 76 | Genes annotated by the GO term GO:0030182 | 6 | 0.0789 | 3.46E-06 | 1.20E-03 |
| GENERATION_OF_NEURONS | 83 | Genes annotated by the GO term GO:0048699 | 6 | 0.0723 | 5.78E-06 | 1.79E-03 |
| CELLULAR_MORPHOGENESIS_DURING_ DIFFERENTIATION | 49 | Genes annotated by the GO term GO:0000904 | 5 | 0.102 | 6.51E-06 | 1.81E-03 |
| NEURITE_DEVELOPMENT | 53 | Genes annotated by the GO term GO:0031175 | 5 | 0.0943 | 9.63E-06 | 2.44E-03 |
| NEUROGENESIS | 93 | Genes annotated by the GO term GO:0022088 | 6 | 0.0645 | 1.12E-05 | 2.59E-03 |
| REACTOME_POTASSIUM_CHANNELS | 98 | Genes involved in Potassium channels | 6 | 0.0612 | 1.51E-05 | 3.23E-03 |
| NEURON_DEVELOPMENT | 61 | Genes annotated by the GO term GO:0048666 | 5 | 0.082 | 1.93E-05 | 3.83E-03 |
| INTRINSIC_TO_PLASMA_MEMBRANE | 991 | Genes annotated by the GO term GO:0031226 | 16 | 0.0161 | 9.65E-05 | 1.79E-02 |
| SYNAPTOGENESIS | 18 | Genes annotated by the GO term GO:0007416 | 3 | 0.1667 | 1.15E-04 | 2.00E-02 |
| INTRINSIC_TO_MEMBRANE | 1348 | Genes annotated by the GO term GO:0031224 | 19 | 0.0141 | 1.29E-04 | 2.11E-02 |
| SYNAPSE_ORGANIZATION_AND_BIOGENESIS | 23 | Genes annotated by the GO term GO:0050808 | 3 | 0.1304 | 2.45E-04 | 3.79E-02 |
| PLASMA_MEMBRANE | 1426 | Genes annotated by the GO term GO:0005886 | 19 | 0.0133 | 2.61E-04 | 3.79E-02 |
| INTEGRAL_TO_PLASMA_MEMBRANE | 977 | Genes annotated by the GO term GO:0005887 | 15 | 0.0154 | 2.72E-04 | 3.79E-02 |
