# Supplementary material for: Reprogramming human gallbladder cells into insulin-producing β-like cells
Source: PLoS One. 2017 Aug 16;12(8):e0181812. doi: 10.1371/journal.pone.0181812 (PMC5558938; doi:10.1371/journal.pone.0181812)
Supplement: S8 Table — (DOCX) [file pone.0181812.s014.docx]

**S8 Table. Gene set investigation of the top 119 “GB genes” upregulated in Hpi1+ rGBC (log_2_FC>5, *p*<0.01) to determine the molecular signatures of gene sets characteristic of unreprogrammed GBC potentially needed to be downregulated for a more efficient genetic reprogramming to beta cells.**

| **Gene Set Name** | **#Genes in Gene Set (K)** | **Description** | **#Genes in Overlap (k)** | **k/K** | **p-value** | **FDR q-value** |
| --- | --- | --- | --- | --- | --- | --- |
| NABA_MATRISOME | 1028 | Ensemble of genes encoding extracellular matrix and extracellular matrix-associated proteins | 24 | 0.0233 | 1.79E-16 | 4.99E-13 |
| NABA_MATRISOME_ASSOCIATED | 753 | Ensemble of genes encoding ECM-associated proteins | 19 | 0.0252 | 8.62E-14 | 1.20E-10 |
| MEMBRANE | 1994 | Genes annotated by the GO term GO:0016020 | 28 | 0.014 | 1.36E-13 | 1.27E-10 |
| PLASMA_MEMBRANE | 1426 | Genes annotated by the GO term GO:0005886 | 22 | 0.0154 | 1.38E-11 | 9.59E-09 |
| MEMBRANE_PART | 1670 | Genes annotated by the GO term GO:0044425 | 22 | 0.0132 | 2.76E-10 | 1.54E-07 |
| PLASMA_MEMBRANE_PART | 1158 | Genes annotated by the GO term GO:0044459 | 18 | 0.0155 | 1.08E-09 | 5.00E-07 |
| INTEGRAL_TO_PLASMA_MEMBRANE | 977 | Genes annotated by the GO term GO:0005887 | 15 | 0.0154 | 3.48E-08 | 1.38E-05 |
| INTRINSIC_TO_PLASMA_MEMBRANE | 991 | Genes annotated by the GO term GO:0031226 | 15 | 0.0151 | 4.19E-08 | 1.46E-05 |
| INTEGRAL_TO_MEMBRANE | 1330 | Genes annotated by the GO term GO:0016021 | 17 | 0.0128 | 5.65E-08 | 1.75E-05 |
| INTRINSIC_TO_MEMBRANE | 1348 | Genes annotated by the GO term GO:0031224 | 17 | 0.0126 | 6.84E-08 | 1.90E-05 |
| REACTOME_HS_GAG_DEGRADATION | 20 | Genes involved in HS-GAG degradation | 4 | 0.2 | 1.94E-07 | 4.78E-05 |
| NABA_ECM_REGULATORS | 238 | Genes encoding enzymes and their regulators involved in the remodeling of the ECM | 8 | 0.0336 | 2.06E-07 | 4.78E-05 |
| REACTOME_A_TETRASACCHARIDE_LINKER_SEQUENCE_IS_REQUIRED_FOR_GAG_ SYNTHESIS | 25 | Genes involved in A tetrasaccharide linker sequence is required for GAG synthesis | 4 | 0.16 | 5.01E-07 | 1.00E-04 |
| CYTOPLASM | 2131 | Genes annotated by the GO term GO:0005737 | 20 | 0.0094 | 5.04E-07 | 1.00E-04 |
| REACTOME_HS_GAG_BIOSYNTHESIS | 31 | Genes involved in HS-GAG biosynthesis | 4 | 0.129 | 1.23E-06 | 2.29E-04 |
| EXTRACELLULAR_REGION | 447 | Genes annotated by the GO term GO:0005576 | 9 | 0.0201 | 2.52E-06 | 4.30E-04 |
| KEGG_ECM_RECEPTOR_INTERACTION | 84 | ECM-receptor interation | 5 | 0.0595 | 2.69E-06 | 4.30E-04 |
| CYTOPLASMIC_PART | 1383 | Genes annotated by the GO term GO:0044444 | 15 | 0.0108 | 2.78E-06 | 4.30E-04 |
| ION_BINDING | 273 | Genes annotated by the GO term GO:0043167 | 7 | 0.0256 | 7.27E-06 | 1.06E-03 |
| CALCIUM_ION_BINDING | 104 | Genes annotated by the GO term GO:0005509 | 5 | 0.0481 | 7.69E-06 | 1.06E-03 |
